# Childhood adversity and health: The mediating roles of emotional expression and general trust

**DOI:** 10.3389/fpsyg.2024.1493421

**Published:** 2024-11-20

**Authors:** Hiroki Hirano, Keiko Ishii

**Affiliations:** Department of Cognitive and Psychological Sciences, Graduate School of Informatics, Nagoya University, Nagoya, Japan

**Keywords:** childhood adversity, emotional expression, general trust, happiness, loneliness, culture

## Abstract

**Introduction:**

This study examined whether adverse childhood experiences, positive emotional expressivity in personal (i.e., expressing positive emotions when good things happened to *oneself*) and social settings (i.e., expressing positive emotions when good things happened to *others* such as friends or family), and general trust predict levels of happiness and loneliness among American and Japanese participants. We also explored whether these two types of emotional expression and general trust mediate the relationship between adverse childhood experiences and happiness/loneliness.

**Methods:**

American and Japanese participants who agreed to participate in the current study first completed the Subjective Happiness Scale. Next, they answered the Emotion Expression Questionnaire, the 5-item General Trust Scale, and the revised UCLA Loneliness Scale. They then responded to the Risky Family Questionnaire. Finally, they answered demographic questions (e.g., age, gender). We hypothesized that regardless of culture, adverse childhood experiences would be negatively (positively) associated with happiness (loneliness), while positive emotional expression in personal and social settings and general trust would be positively (negatively) related to happiness (loneliness). We also predicted that positive emotional expression in both personal and social settings, as well as general trust, would mediate the relationships between adverse childhood experiences and happiness/loneliness.

**Results:**

As expected, adverse childhood experiences were negatively (positively) associated with happiness (loneliness), while positive emotional expression in personal and social settings and general trust were positively (negatively) related to happiness (loneliness). Besides, positive emotional expression in a personal situation mediated the relationships between adverse childhood experiences and happiness/loneliness, such that greater early life adversity was negatively linked to positive emotional expressivity in a personal setting, which, in turn, predicted lower happiness and higher loneliness.

**Discussion:**

The present study advances the understanding of psychological mechanisms linking adverse childhood experiences to happiness and loneliness by highlighting the significant role of positive emotional expression in a personal situation. This result underscores the importance of developing therapeutic practices and public health strategies that foster authentic emotional expression in response to personal achievement or fortune, regardless of cultural background.

## 1 Introduction

A number of previous studies have substantiated that adverse childhood experiences, which include a range of traumatic experiences such as violence, emotional abuse, and neglect from caregivers, disrupt developmental processes and lead to both immediate and long-term repercussions on physical and psychological health (e.g., Balistreri and Alvira-Hammond, 2016; Ohtsubo et al., 2021; Wang et al., 2024). What is less known, however, is how psychological processes, particularly *emotional expression* and *general trust*, play roles in these relationships. In general, those who experienced childhood maltreatment are more likely to adopt maladaptive emotion regulation strategies (e.g., rumination, emotional suppression; Miu et al., 2022) and have lower levels of trust in others (Zheng et al., 2020), both of which increase susceptibility to psychopathology. Nevertheless, a systematic understanding of how early life adversity influences emotional expressivity and trust-related behaviors, as well as their impact on psychological experiences such as happiness and loneliness (which are also closely linked to mental health outcomes), is still lacking and warrants further investigation (Milojevich et al., 2021; Zheng et al., 2020). Therefore, the primary objective of the current study is to investigate and elucidate these associations across two different cultural contexts: Japan and the United States.

### 1.1 Adverse childhood experiences and implications for health outcomes

Adverse childhood experiences are generally defined as “potentially traumatic events that can have negative lasting effects on health and wellbeing” (Boullier and Blair, 2018, p. 132). Although not exhaustive, these events include physical, emotional, and sexual abuse, neglect, and household dysfunction, such as witnessing domestic violence in the household (Boullier and Blair, 2018). Prior research has demonstrated that adverse childhood experiences have a significant impact on developmental trajectories, contributing to both mental and physical health issues. For example, individuals with a history of childhood maltreatment report reduced life satisfaction and lower subjective, psychological, and social well-being (Mosley-Johnson et al., 2019; Ohtsubo et al., 2021), and are at higher risk for anxiety, depression, suicidal ideation, and loneliness (Afifi et al., 2008; Chapman et al., 2004; Merz and Jak, 2013; Ohtsubo et al., 2021). Early life adversity also disrupts physiological and behavioral stability, a process known as *allostasis* (Boullier and Blair, 2018). When faced with stress, the body’s sympathetic nervous system and hypothalamic–pituitary–adrenal (HPA) axis are activated, releasing cortisol to prepare the body for a “fight or flight” response. However, chronic or repeated stress without receiving support from caregivers can lead to dysregulation of these allostatic systems, causing long-term damage to neurological, endocrine, and immune functions. This, in turn, results in altered cortisol production and chronic inflammation, which increase the risk of infections and cardiovascular diseases (Boullier and Blair, 2018). Besides, adverse childhood experiences are associated with higher rates of health risk behaviors, including alcoholism, drug abuse, smoking, physical inactivity, overeating, and unprotected sexual intercourse (Campbell et al., 2016; Felitti et al., 1998), all of which impair adaptive functioning. Taken together, the effects of early life adversity may extend well beyond childhood, influencing both physical and psychological health outcomes across the lifespan (Bellis et al., 2013; Kessler et al., 2010).

### 1.2 Emotional expression and adverse childhood experiences

Previous research has shown that emotional expression, conceptualized as an outward manifestation of an intrapsychic state through both verbal and non-verbal behavior (e.g., vocal tone, facial expression), is regarded as an important part of human functioning (Skinner, 2013). For instance, emotional expression has been associated with lower negative affect (Stanton et al., 2000a) and improved physical/psychological adjustment (Stanton et al., 2000b). Expressing emotion (e.g., articulating emotions) can also help individuals recognize, understand, and interpret their internal states, thereby providing valuable insights into their experiences (e.g., emotional epiphanies; Kennedy-Moore and Watson, 2001). Moreover, those who express emotions are more likely to have intimate relationships with others (Graham et al., 2008), in part because they are perceived as more authentic (Mauss et al., 2011) and trustworthy (Boone and Buck, 2003). In line with these findings, *expressive suppression*, which refers to a form of emotion regulation where an individual consciously *inhibits* the outward expression of their emotions (Gross and John, 2003), has been linked to maladaptive functioning such as lower happiness (Hirano and Ishii, 2024; Kelley et al., 2019) and increased risk of psychopathology (Dryman and Heimberg, 2018) as well as loneliness (Hirano and Ishii, 2024; Preece et al., 2021). It should be pointed out, however, that suppressing emotions can be beneficial in some circumstances (e.g., maintaining harmonious interpersonal relationships; Soto et al., 2016), suggesting that its effectiveness may depend on the context (Kashdan and Rottenberg, 2010). That said, prior literature generally suggests that expressing genuine emotion appears to be particularly crucial for promoting psychological health.

Early life experiences lay the foundation for how individuals regulate emotions later in life (Silvers, 2022). Specifically, childhood adversity is associated with maladaptive emotion regulation strategies such as expressive suppression and rumination (Miu et al., 2022). Chronic stress from adverse childhood experiences leads to HPA axis dysfunction (Kalmakis et al., 2015), which can negatively impact emotional development (Lupien et al., 2009) and undermine cognitive functioning, including reduced emotion regulation skills (Raymond et al., 2018). Similarly, individuals raised in unstable or harmful environments often develop insecure attachment styles because of inconsistent and negative caregiving experiences (Iwaniec et al., 2007), which impairs the ability to regulate emotions effectively (Mikulincer and Shaver, 2019). Furthermore, maltreating parents often report having difficulties controlling their emotions (Lavi et al., 2019), and because children mostly learn coping strategies from their caregivers through social learning (Morris et al., 2017), they may internalize and replicate these maladaptive patterns in their own emotional responses (Milojevich et al., 2020). Accordingly, it is expected that people who have experienced childhood adversity are more likely to have difficulty expressing their emotions. However, it is somewhat surprising that there is a paucity of research examining these relationships (Milojevich et al., 2021). Therefore, in the present investigation, we examine how early life experiences shape emotional expressivity and ultimately affect health outcomes. To be more specific, we purposely focus on *positive* emotional expression (PEE) in situations where something good happened either personally (PEE-P) or socially (PEE-S; e.g., friends, family members). Previous studies have noted that expressing positive emotion is beneficial from both intra- and interpersonal perspectives. For instance, individuals who express genuine positive emotions are more likely to experience higher wellbeing than those who do not, partly due to the consistency between their internal states and external expressions, along with increased social connectedness (English and John, 2013; Mauss et al., 2011). The happiness of close others, such as friends (i.e., the context of PEE-S), is also related to one’s own better mental state (Matsunaga et al., 2017). Moreover, people who express positive emotions are rated more positively by others in various domains, including likability, warmth, friendliness, and intelligence (Lyubomirsky et al., 2005). Thus, by examining these two situationally distinct yet interconnected forms of PEE, we aim to gain a deeper understanding of how adverse childhood experiences impact later-life psychological health.

### 1.3 Trust and adverse childhood experiences

Although the term *trust* can be defined in specific ways (e.g., institutional trust), its fundamental conceptualization is “the willingness of the trustor to become vulnerable to the trustee” (Schilke et al., 2021, p. 240). Previous literature has shown that trust is associated with a range of physical and psychological health benefits. For instance, people with higher baseline levels of trust exhibit better physical health (Barefoot et al., 1998), greater life satisfaction (Bjørnskov, 2006)and wellbeing (Jovanović, 2016), and a decreased risk of suicide (Benson et al., 2016). Communities and nations with greater levels of trust navigate and deal with transitions (e.g., economic and financial crises) in a more positive and efficient way (Helliwell et al., 2014). Although the mechanisms through which trust influences wellbeing are multifaceted, one key factor is the formation of social support networks. Trust promotes cooperative attitudes, which helps develop positive relationships with others (Tov and Diener, 2009). As such, trusting individuals are more likely to receive social resources, which is a critical component of adaptive functioning (Siedlecki et al., 2014). By contrast, distrust of others hinders the development of such networks, resulting in maladaptive functioning (e.g., greater loneliness; Nyqvist et al., 2016). It is worth emphasizing, however, that trust is not without risks; it can lead to negative outcomes such as exploitation by others, suggesting that a healthy degree of distrust can still be functional (Scharte, 2024). Nevertheless, as explained above, trust in others generally plays a foundational role in interpersonal relationships and carries significant implications for health outcomes.

Early life adversity is linked to lower levels of trust (Brown, 2023; Zheng et al., 2020). Central to this relationship is the role of parenting, especially the development of insecure attachment styles (Stolle, 2002; Iwaniec et al., 2007). Those who experience greater adversity are more likely to develop disorganized attachment styles (e.g., avoidant or anxious attachment) because their relationships with primary caregivers are severely disrupted during critical developmental periods. This leads to maladaptive beliefs and expectations about others, which in turn contribute to lower levels of trust (Mikulincer, 1998). Given that generalized trust tends to stabilize prior to adulthood (Wu, 2021), adverse childhood experiences can give rise to ongoing difficulties in establishing and maintaining meaningful and supportive interpersonal relationships later in life. Harsh childhood environments are also associated with faster life history strategies (i.e., prioritizing immediate rewards over long-term gains; Griskevicius et al., 2011), which may foster a sense of mistrust toward others (Wu et al., 2017). Moreover, adverse childhood experiences may alter neurobiological mechanisms and hormonal activities (Dye, 2018; Teicher and Samson, 2016; but see also Riedl and Javor, 2012), leading to diminished trust in social contexts (e.g., Bos et al., 2010; Koscik and Tranel, 2011). Overall, these findings indicate that adverse childhood experiences have significant implications for the development of trust (Brown, 2023). Nevertheless, it is worth highlighting that there have been relatively few attempts to directly investigate the impact of early life experiences on trusting behaviors (Zheng et al., 2020). Thus, in the current analyses, we also explore how trust plays a role in the link between childhood maltreatment and human functioning. In particular, we adopt the concept of *general* trust. General trust is one of the most common forms examined in previous literature (e.g., Zheng et al., 2020) and is defined as “a belief in the benevolence of human nature in general” (Yamagishi and Yamagishi, 1994, p. 139). Consistent with the definition of trust introduced in the previous paragraph, general trust is a default trust placed in others when there is not enough information available to determine their trustworthiness. We opted for general trust because it underpins everyday social interactions and is associated with greater happiness (Jasielska et al., 2021). Put differently, measuring general trust allows us to probe into a fundamental perspective on how individuals approach and interpret their social environment, which is closely linked to adaptive functioning. This approach therefore elucidates critical insights into how early life experiences influence broader social attitudes and impact long-term psychological health.

### 1.4 Overview of the current study

Although adverse childhood experiences appear to have significant implications for emotional expression and the formation of trust in others, little research (e.g., Ohtsubo et al., 2021) has directly examined these relationships as well as their impact on mental health outcomes. Considering the long-lasting effects of early life experiences on human functioning, identifying the underlying mechanisms (e.g., mediating variables) becomes imperative in order to develop more effective interventions (Cloitre et al., 2019). Therefore, the current study aimed to fill this gap by investigating the pathways through which early life adversity influences positive emotional expression in personal/social settings and general trust, and how these factors in turn affect levels of happiness and loneliness. We selected happiness as a dependent variable because it serves as a reliable indicator of human functioning (Lyubomirsky et al., 2005), making it a valuable measure for assessing the impact of adverse childhood experiences on psychological health. Similarly, we measured loneliness as it captures the quality of interpersonal relationships and is associated with psychopathology (Cacioppo and Cacioppo, 2014). Given the relational nature of trust and emotional expression, particularly in a social setting, incorporating loneliness into the model thus provides deeper insights into how early life experiences affect social functioning in addition to psychological health.

In the current analyses, we specifically targeted American participants (Study 1) and Japanese participants (Study 2). These two nations were selected because they represent distinct cultural orientations (e.g., individualism and collectivism; Markus and Kitayama, 1991), which helps enhance the generalizability of our findings. Besides, well-established translations (i.e., English and Japanese versions) of each measure were available from prior research, making data collection feasible while ensuring cross-cultural validity. This is critical, given that previous studies examining the impact of childhood adversity on wellbeing in different cultural settings have often relied on publicly available datasets such as the Midlife in the United States (MIDUS; e.g., Kong et al., 2021), and thus collecting new data with underexplored variables (e.g., emotional expression and general trust) allows our study to serve as an important external validation of prior findings and helps extend the understanding of potential underlying mechanisms.

#### 1.4.1 Hypotheses

Although culture exerts a unique influence on thoughts, cognition, and behavior (Markus and Kitayama, 1991), prior research involving individuals from East Asia (e.g., Japan, China) and North America (e.g., Canada, the United States) implies that the negative impact of early life adversity on trust and emotion regulation (e.g., diminished trust, impaired emotion regulation skills), as well as their adverse effects on physical/psychological health, appear to be consistent across these cultures (e.g., Cloitre et al., 2019; Ohtsubo et al., 2021; Rudenstine et al., 2019; Ye et al., 2024; Zheng et al., 2020). Therefore, we hypothesized that regardless of culture, adverse childhood experiences would be negatively associated with happiness, while PEE-P/PEE-S and general trust would be positively related to happiness (Hypothesis 1). On the other hand, adverse childhood experiences would be positively associated with loneliness, whereas PEE-P/PEE-S and general trust would be negatively linked to loneliness (Hypothesis 2). Similarly, we expected that regardless of culture, PEE-P/PEE-S and general trust would mediate the relationship between adverse childhood experiences and happiness, with harsher childhood environments predicting lower PEE-P/PEE-S and general trust, leading to lower levels of happiness (Hypothesis 3). Similarly, we hypothesized that PEE-P/PEE-S and general trust would mediate the relationship between adverse childhood experiences and loneliness, with greater adversity being associated with lower levels of PEE-P/PEE-S and general trust, which would, in turn, predict greater loneliness (Hypothesis 4).

#### 1.4.2 Ethics statement and openness

The current study received ethical approval from Nagoya University (NUPSY-240709-M-01). We report how we determined our sample size, data exclusions (if any), and all hypotheses (see pre-registration for more details: https://osf.io/rne5q). Due to the nature of web surveys, oral/written consent signatures could not be obtained. However, participants were informed at the beginning of the survey that their responses would remain anonymous and confidential, and that they were not obligated to answer any questions they were uncomfortable with. They were also free to withdraw from participation at any time or choose not to participate at all, without any penalties or loss of benefits to which they were otherwise entitled. By agreeing to these terms, it is considered that they have consented to participate in the study.

## 2 Study 1

### 2.1 Methods

#### 2.1.1 Participants

A power analysis was conducted using G*Power (Faul et al., 2007) to determine the necessary sample size for our study. To detect a medium effect size (*f*^2^ = 0.15) with 95% power in an F test (Linear multiple regression: Fixed model, R^2^ deviation from zero) with three predictors—childhood family environment, (either) PEE-P or PEE-S, and general trust—and a significance level of 0.05, a minimum of 119 participants was required. To account for potential data screening and ensure a sufficiently large sample, we recruited 205 American participants through an online research platform Prolific. After data cleaning, seven participants who failed to answer more than 50% of the items on a particular measure were removed (no data were deleted based on response time, duplicate IP addresses, or failure to answer an attention check question), leading the data of 198 participants to be analyzed (121 female participants, *M*_age_ = 38.70, *SD* = 12.51, 99.5% White).

#### 2.1.2 Procedure

Participants who agreed to participate in the current study first completed the Subjective Happiness Scale (Lyubomirsky and Lepper, 1999). Next, they answered the Emotion Expression Questionnaire (Salvador et al., 2024), the 5-item General Trust Scale (Yamagishi et al., 2015), and the revised UCLA Loneliness Scale (Russell et al., 1980). They then responded to the Risky Family Questionnaire (Taylor et al., 2004). Finally, they answered demographic questions (e.g., age, gender).

#### 2.1.3 Measures

##### 2.1.3.1 Happiness

Happiness was assessed with the Subjective Happiness Scale (a 4-item scale; Lyubomirsky and Lepper, 1999). In the first two items, participants were asked to evaluate their absolute happiness (I consider myself: 1 = *not a very happy person* to 7 = *a very happy person*) and relative happiness (Compared to most of my peers, I consider myself: 1 = *less happy* to 7 = *more happy*). The other two items describe generally happy and unhappy individuals (e.g., Some people are generally very happy. They enjoy life regardless of what is going on, getting the most out of everything), and participants rated how closely these descriptions applied to them (1 = *not at all* to 7 = *a great deal*). The alpha reliability of the scale was *α* = 0.91. All scale items are included in the Supplementary material.

##### 2.1.3.2 Positive emotional expression

The Emotion Expression Questionnaire (Salvador et al., 2024) was adopted to measure participants’ tendencies to express seven different positive emotions (e.g., happy). Specifically, we measured positive emotional expression in a situation where good things happened either personally (PEE-P; e.g., When you have succeeded in an exam or assignment, how strongly would you express “happy” when you discuss your experience with your friends or family members?) or socially (PEE-S; e.g., When you learned about something good that happened to your friends or family, How strongly would you express “happy” when you discuss your experience with your friends or family members?). Put differently, PEE-P involves expressing positive emotions in response to one’s own achievements or good fortune and is more self-oriented, while PEE-S is more outwardly focused, reflecting positive emotional expression when others, such as friends or family, experience success or happiness. We purposefully separated personal and social situations because, although both involve positive emotional expression and benefit mental health, the ways in which these forms of expressivity are influenced by early life adversity may vary—especially considering cultural differences in the emphasis on autonomy versus attunement to interpersonal relationships (Markus and Kitayama, 1991; Triandis, 1989). Therefore, by accounting for these situational factors, we aimed to capture more nuanced aspects of emotional expressivity.^1^ Each emotion was rated on a 6-point Likert scale of 1 (*Not at all*) to 6 (*Very strongly*). The alpha reliability of PEE-P and PEE-S was *α* = 0.83 and *α* = 0.85, respectively. The full set of scale items is provided in the Supplementary material.

##### 2.1.3.3 General trust

An English version of the 5-item General Trust Scale (Yamagishi et al., 2015), originally developed and validated by Yamagishi and Yamagishi (1994) and then partly modified by Yamagishi et al. (2013), was adopted to assess participants’ beliefs in the trustworthiness of others. (e.g., “Most people are basically good and kind”). Participants rated each item on a 7-point Likert scale, from 1 (*Strongly disagree*) to 7 (*Strongly agree*). The alpha reliability of the scale was *α* = 0.93. Details of all scale items can be found in the Supplementary material.

##### 2.1.3.4 Loneliness

The revised UCLA Loneliness Scale (a 20-item scale; Russell et al., 1980) was used to measure subjective feelings of loneliness and social isolation (e.g., I feel in tune with the people around me). Each item was rated on a 4-point Likert scale of 1 (*Never*) to 4 (*Often*). The alpha reliability of the scale was *α* = 0.96. All the items from the scale are available in the Supplementary material.

##### 2.1.3.5 Adverse childhood experiences

Early life experiences were evaluated with the Risky Family Questionnaire (Taylor et al., 2004), which includes 13 items related to adverse childhood experiences (e.g., “How often did a parent or other adult in the household swear at you or act in a way that made you feel threatened?”). Participants rated each item on a 5-point Likert scale, ranging from 1 (*Not at all*) to 5 (*Very often*). The alpha reliability of the scale was *α* = 0.92. All items from the scale are provided in the Supplementary material.

#### 2.1.4 Data analysis

To test Hypotheses 1 and 2, multiple regression analyses were conducted with adverse childhood experiences, positive emotional expression (i.e., PEE-P or PEE-S), and general trust as the independent variables, and happiness and loneliness as the dependent variables. With regard to Hypotheses 3 and 4, mediation analyses were adopted to investigate whether PEE-P or PEE-S and general trust would mediate the relationship between adverse childhood experiences and happiness/loneliness. Because all variables were normally distributed (e.g., skewness and kurtosis values fell within the acceptable range; Hair et al., 2022), no data transformations were performed (for more details, see Supplementary Table S1). Given the mediation analysis testing the sets of two relative direct, indirect, and total effects, a *p*-value criterion of 0.025 and 97.5% CI, instead of 0.05 and 95% CI, were adopted to reduce the risk of type I error (Hayes and Preacher, 2014). Although our pre-registration and power analysis determined the sample size based on a significance level of 0.05, the current sample size remains sufficient to meet the adjusted criterion of 0.025 (for more details, see the Supplementary material).

### 2.2 Results

#### 2.2.1 Descriptive statistics and correlations

Table 1 presents means, standard deviations, and correlations between each variable. Adverse childhood experiences were negatively associated with PEE-P (*r* = −0.22, *p* = 0.002), general trust (*r* =−0.15, *p* = 0.03), and happiness (*r* = −0.25, *p* < 0.001), and positively correlated with loneliness (*r* = 0.29, *p* < 0.001). PEE-P, PEE-S, and general trust were also positively related to happiness (0.37 < *r*s < 0.42, *p*s < 0.001) and negatively linked to loneliness (−0.49 < *r*s < −0.35, *p*s < 0.001).

**Table 1:** Descriptive statistics and correlations among American participants (Study 1).

	*M*	*SD*	1	2	3	4	5	6
1. ACEs	2.43	0.93	–					
2. PEE-P	4.14	0.95	−0.22**	–				
3. PEE-S	4.31	0.91	−0.13	0.61***	–			
4. Trust	4.05	1.31	−0.15*	0.27***	0.32***	–		
5. Happiness	4.29	1.36	−0.25***	0.39***	0.37***	0.42***	–	
6. Loneliness	43.85	14.46	0.29***	−0.49***	−0.41***	−0.35***	−0.67***	–

*p < 0.05; **p < 0.01; ***p < 0.001.M, mean; SD, standard deviation; ACEs, adverse childhood experiences; PEE-P, positive emotional expression in a personal situation; PEE-S, positive emotional expression in a social situation.

#### 2.2.2 The influence of adverse childhood experiences, PEE-P/PEE-S, and trust on happiness (Hypothesis 1)

Multiple regression was conducted to test if adverse childhood experiences, PEE-P, and general trust would predict happiness. Results demonstrated that the overall regression was significant [*R^2^* = 0.28, *F* (3, 194) = 25.21, *p* < 0.001]. Adverse childhood experiences were not significantly associated with happiness (*β* = −0.14, *p* = 0.03, 97.5% CI [−0.28, 0.001]), while higher PEE-P (*β* = 0.27, *p* < 0.001, 97.5% CI [0.12, 0.42]) and general trust (*β* = 0.33, *p* < 0.001, 97.5% CI [0.19, 0.47]) predicted greater happiness.

Next, we tested if adverse childhood experiences, PEE-S, and general trust would predict happiness. Results showed that the overall regression was significant [*R^2^* = 0.27, *F* (3, 194) = 24.13, *p* < 0.001]. Greater adverse childhood experiences were associated with lower happiness (*β* = −0.17, *p* = 0.01, 97.5% CI [−0.31, −0.03]), whereas higher PEE-S (*β* = 0.25, *p* < 0.001, 97.5% CI [0.11, 0.40]) and general trust (*β* = 0.32, *p* < 0.001, 97.5% CI [0.17, 0.47]) predicted greater happiness.

#### 2.2.3 The influence of adverse childhood experiences, PEE-P/PEE-S, and trust on loneliness (Hypothesis 2)

First, we tested whether adverse childhood experiences, PEE-P, and general trust would predict loneliness. Results demonstrated that the overall regression model was significant [*R*^2^ = 0.32, *F* (3, 194) = 30.92, *p* < 0.001]. Greater adverse childhood experiences were associated with higher loneliness (*β* = 0.17, *p* = 0.005, 97.5% CI [0.04, 0.31]), while higher scores in PEE-P (*β* = −0.40, *p* < 0.001, 97.5% CI [−0.54, −0.26]) and general trust (*β* = −0.22, *p* = 0.001, 97.5% CI [−0.36, −0.08]) predicted lower loneliness.

Next, we explored whether adverse childhood experiences, PEE-S, and general trust would predict loneliness. Results showed that the overall regression model was significant [*R*^2^ = 0.27, *F* (3, 194) = 23.58, *p* < 0.001]. Adverse childhood experiences predicted greater loneliness (*β* = 0.22, *p* < 0.001, 97.5% CI [0.08, 0.36]), even though higher PEE-S (*β* = −0.31, *p* < 0.001, 97.5% CI [−0.45, −0.16]) and general trust (*β* = −0.22, *p* < 0.001, 97.5% CI [−0.37, −0.07] were linked to lower loneliness.

#### 2.2.4 Mediation analyses for happiness (Hypothesis 3)

**Figure 1:**
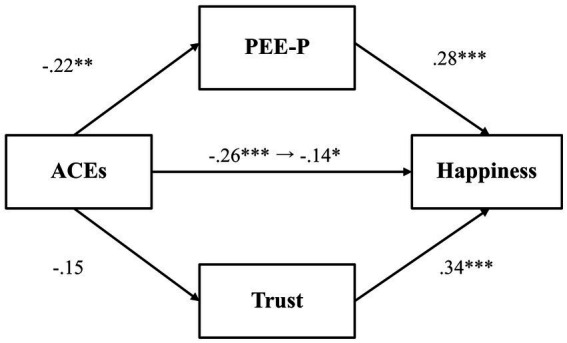
The mediating effect of PEE-P and trust in the link between ACEs and happiness (Study 1). *p < 0.025, **p < 0.01, and ***p < 0.001. ACEs, adverse childhood experiences; PEE-P, positive emotional expression in a personal situation. All values represent standardized coefficients.

A mediation analysis was conducted to first explore whether PEE-P and general trust would mediate the relationship between adverse childhood experiences and happiness (Figure 1). Results indicated that adverse childhood experiences were negatively associated with PEE-P (*β* = −0.22, *p* = 0.001, 97.5% CI [−0.37, −0.07]), and PEE-P, in turn, positively predicted happiness (*β* = 0.28, *p* < 0.001, 97.5% CI [0.14, 0.41]). The indirect effect of PEE-P (*β* = −0.06, *p* = 0.01, 97.5% CI [−0.11, −0.01]) was also significant. Adverse childhood experiences, by contrast, did not predict general trust (*β* = −0.15, *p* = 0.03, 97.5% CI [−0.31, 0.003]), even though general trust was positively associated with happiness (*β* = 0.34, *p* < 0.001, 97.5% CI [0.21, 0.47]). The total effect (*β* = −0.26, *p* < 0.001, 97.5% CI [−0.40, −0.11]) and the direct effect (*β* = −0.14, *p* = 0.02, 97.5% CI [−0.29, −0.002]) were both significant, indicating that even after accounting for the mediator variables, there was a significant association between adverse childhood experiences and happiness.

**Figure 2:**
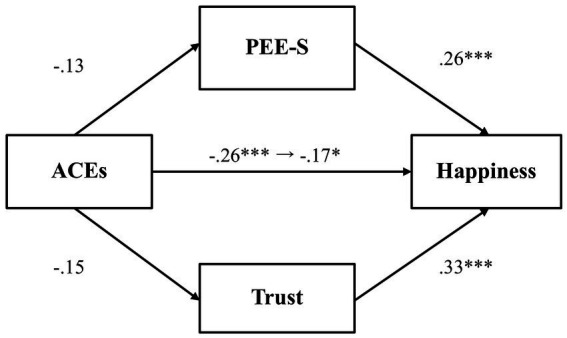
The mediating effect of PEE-S and trust in the link between ACEs and happiness (Study 1). *p < 0.025, **p < 0.01, and ***p < 0.001. ACEs, adverse childhood experiences; PEE-S, positive emotional expression in a social situation. All values represent standardized coefficients.

Next, we tested whether PEE-S and general trust would mediate the relationship between adverse childhood experiences and happiness (Figure 2). Results demonstrated that there was no significant relationship between adverse childhood experiences and PEE-S (*β* = −0.13, *p* = 0.07, 97.5% CI [−0.28, 0.03]), while PEE-S was positively related to happiness (*β* = 0.26, *p* < 0.001, 97.5% CI [0.12, 0.39]). Adverse childhood experiences also did not predict general trust (*β* = −0.15, *p* = 0.03, 97.5% CI [−0.31, 0.003]), while general trust was positively associated with happiness (*β* = 0.33, *p* < 0.001, 97.5% CI [0.19, 0.46]). The total effect (*β* = −0.26, *p* < 0.001, 97.5% CI [−0.40, −0.11]) and the direct effect (*β* = −0.17, *p* = 0.01, 97.5% CI [−0.31, −0.04]) were both significant, indicating that even after controlling for the mediator variables, the association between adverse childhood experiences and happiness was significant.

#### 2.2.5 Mediation analyses for loneliness (Hypothesis 4)

**Figure 3:**
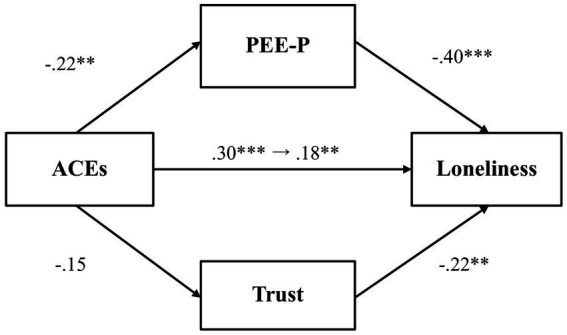
The mediating effect of PEE-P and trust in the link between ACEs and loneliness (Study 1). **p < 0.01 and ***p < 0.001. ACEs, adverse childhood experiences; PEE-P, positive emotional expression in a personal situation. All values represent standardized coefficients.

We first examined whether PEE-P and general trust would mediate the relationship between adverse childhood experiences and loneliness (Figure 3). Results showed that adverse childhood experiences were negatively associated with PEE-P (*β* = −0.22, *p* = 0.001, 97.5% CI [−0.37, −0.07]), and PEE-P, in turn, negatively predicted loneliness (*β* = −0.40, *p* < 0.001, 97.5% CI [−0.53, −0.28]). The indirect effect of PEE-P (*β* = 0.09, *p* = 0.004, 97.5% CI [0.02, 0.16]) was also significant. By contrast, adverse childhood experiences did not predict general trust (*β* = −0.15, *p* = 0.03, 97.5% CI [−0.31, 0.003]), although general trust was negatively associated with loneliness (*β* = −0.22, *p* < 0.001, 97.5% CI [−0.36, −0.09]). The total effect (*β* = 0.30, *p* < 0.001, 97.5% CI [0.16, 0.44]) and the direct effect (*β* = 0.18, *p* = 0.004, 97.5% CI [0.04, 0.31]) were both significant, demonstrating that even after accounting for the mediator variables, there was still a significant association between adverse childhood experiences and loneliness.

**Figure 4:**
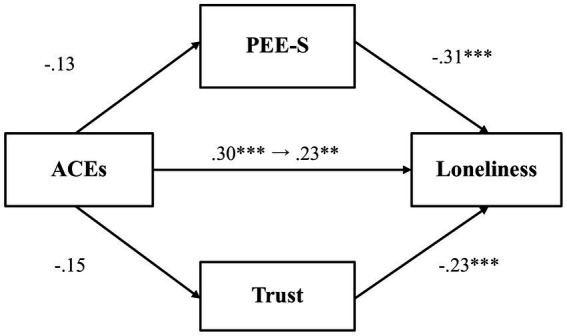
The mediating effect of PEE-S and trust in the link between ACEs and loneliness (Study 1). **p < 0.01 and ***p < 0.001. ACEs, adverse childhood experiences; PEE-S, positive emotional expression in a social situation. All values represent standardized coefficients.

We then investigated whether PEE-S and general trust would mediate the relationship between adverse childhood experiences and loneliness (Figure 4). Results showed that adverse childhood experiences did not predict PEE-S (*β* = −0.13, *p* = 0.07, 97.5% CI [−0.28, 0.03]), even though PEE-S was negatively associated with loneliness (*β* = −0.31, *p* < 0.001, 97.5% CI [−0.45, −0.18]). Likewise, adverse childhood experiences were not significantly associated with general trust (*β* = −0.15, *p* = 0.03, 97.5% CI [−0.31, 0.003]), even though general trust predicted lower loneliness (*β* = −0.23, *p* < 0.001, 97.5% CI [−0.36, −0.09]). The total effect (*β* = 0.30, *p* < 0.001, 97.5% CI [0.16, 0.44]) and the direct effect (*β* = 0.23, *p* = 0.001, 97.5% CI [0.09, 0.36]) were both significant, showing that even after accounting for the mediator variables, there remains a significant association between adverse childhood experiences and loneliness.

### 2.3 Discussion

In general, as hypothesized, adverse childhood experiences were associated with lower happiness and greater loneliness. In contrast, PEE-P, PEE-S, and general trust were linked to greater happiness and lower loneliness. Moreover, PEE-P mediated the relationship between adverse childhood experiences and happiness/loneliness, even though PEE-S and general trust did not.

## 3 Study 2

Although Study 1 indicated a negative impact of early life adversity, the exclusive focus on American participants may limit the generalizability of the findings to other cultural contexts. Therefore, the primary aim of Study 2 was to conduct the same analyses with a Japanese sample to assess the influence of adverse childhood experiences across cultures.

### 3.1 Methods

#### 3.1.1 Participants

Based on the same rationale as Study 1, we recruited 223 Japanese participants through the online research platform Lancers. After data cleaning, 15 participants who failed to answer more than 50% of the items on a particular measure and 2 participants who failed to answer an attention check question (the same attention check item used in Study 1) were removed (no data were deleted based on response time or duplicate IP addresses), resulting in a final sample of 206 participants (77 female; *M*_age_ = 44.58, *SD* = 9.61) to be analyzed.

#### 3.1.2 Procedure

Study 2 adhered to the same procedure as those applied in Study 1.

#### 3.1.3 Measures

##### 3.1.3.1 Happiness

A Japanese version of the Subjective Happiness Scale, developed and validated by Shimai et al. (2004), was administered to measure respondents’ levels of happiness. The reliability of the scale was *α* = 0.87. All scale items are included in the Supplementary material.

##### 3.1.3.2 Positive emotional expression

A Japanese version of the Emotion Expression Questionnaire, created and validated in the study conducted by Salvador et al. (2024), was administered to assess positive emotional expression in personal (PEE-P) and social (PEE-S) situations. The alpha reliability of PEE-P and PEE-S was *α* = 0.76 and *α* = 0.81, respectively. All items from the scale can be found in the Supplementary material.

##### 3.1.3.3 General trust

A Japanese version of the 5-item General Trust Scale (Yamagishi et al., 2015), originally developed and validated by Yamagishi and Yamagishi (1994) and subsequently modified by Yamagishi et al. (2013), was used to assess participants’ beliefs in the trustworthiness of others. The alpha reliability of the scale in the current study was *α* = 0.93. All items for the scale are provided in the Supplementary material.

##### 3.1.3.4 Loneliness

A Japanese version of the revised UCLA Loneliness Scale, which Moroi (1992) developed and validated its reliability and validity, was used to measure levels of loneliness. The alpha reliability of the scale was *α* = 0.97. All scale items are included in the Supplementary material.

##### 3.1.3.5 Adverse childhood experiences

Early life experiences were evaluated using a Japanese version of the Risky Family Questionnaire, which was created and validated by Zheng et al. (2020). The alpha reliability of this scale was *α* = 0.85. The complete set of scale items can be found in the Supplementary material.

#### 3.1.4 Data analysis

We performed the same analyses as we did in Study 1. All variables were normally distributed (e.g., skewness and kurtosis values were within the acceptable range; Hair et al., 2022), and thus no data transformations were conducted (for more details, see Supplementary Table S1). Consistent with Study 1, a *p*-value criterion of 0.025 and 97.5% CI were adopted.

### 3.2 Results

#### 3.2.1 Descriptive statistics and correlations

Table 2 presents means, standard deviations, and correlations between each variable. Adverse childhood experiences were negatively associated with PEE-P (*r* = −0.21, *p* = 0.002), PEE-S (*r* = −0.16, *p* = 0.02), general trust (*r* = −0.16, *p* = 0.03), and happiness (*r* = −0.23, *p* < 0.001), and positively correlated with loneliness (*r* = 0.37, *p* < 0.001). In contrast, PEE-P, PEE-S, and general trust were positively related to happiness (0.33 < *r*s < 0.39, *p*s < 0.001) and negatively linked to loneliness (−0.49 < *r*s < −0.42, *p*s < 0.001).

**Table 2:** Descriptive statistics and correlations among Japanese participants (Study 2).

	*M*	*SD*	1	2	3	4	5	6
1. ACEs	2.14	0.60	–					
2. PEE-P	3.79	0.78	−0.21**	–				
3. PEE-S	3.78	0.80	−0.16*	0.70***	–			
4. Trust	3.75	1.18	−0.16*	0.26***	0.22**	–		
5. Happiness	3.76	1.27	−0.23***	0.39***	0.33***	0.37***	–	
6. Loneliness	48.74	14.66	0.37***	−0.49***	−0.42***	−0.43***	−0.72***	–

*p < 0.05, **p < 0.01, and ***p < 0.001. M, mean; SD, standard deviation; ACEs, adverse childhood experiences; PEE-P, positive emotional expression in a personal situation; PEE-S, positive emotional expression in a social situation.

#### 3.2.2 The influence of adverse childhood experiences, PEE-P/PEE-S, and trust on happiness (Hypothesis 1)

Multiple regression was conducted to test if adverse childhood experiences, PEE-P, and general trust would predict happiness. Results demonstrated that the overall regression was significant [*R^2^* = 0.25, *F* (3, 202) = 21.98, *p* < 0.001]. Adverse childhood experiences were not significantly associated with happiness (*β* = −0.13, *p* = 0.04, 97.5% CI [−0.27, 0.02]), even though higher PEE-P (*β* = 0.29, *p* < 0.001, 97.5% CI [0.15, 0.44]) and general trust (*β* = 0.28, *p* < 0.001, 97.5% CI [0.13, 0.42]) predicted greater happiness.

Next, we tested if adverse childhood experiences, PEE-S, and general trust would predict happiness. Results showed that the overall regression was significant [*R^2^* = 0.22, *F* (3, 202) = 19.25, *p* < 0.001]. Adverse childhood experiences were negatively associated with happiness (*β* = −0.15, *p* = 0.02, 97.5% CI [−0.29, −0.01]), while higher PEE-S (*β* = 0.24, *p* < 0.001, 97.5% CI [0.10, 0.39]) and general trust (*β* = 0.29, *p* < 0.001, 97.5% CI [0.15, 0.44]) predicted greater happiness.

#### 3.2.3 The influence of adverse childhood experiences, PEE-P/PEE-S, and trust on loneliness (Hypothesis 2)

We first tested if adverse childhood experiences, PEE-P, and general trust would predict loneliness. Results indicated that the overall regression was significant [*R^2^* = 0.40, *F* (3, 202) = 44.62, *p* < 0.001]. Adverse childhood experiences (*β* = 0.24, *p* < 0.001, 97.5% CI [0.11, 0.37]) predicted greater loneliness, while higher PEE-P (*β* = −0.36, *p* < 0.001, 97.5% CI [−0.49, −0.23]) and general trust (*β* = −0.30, *p* < 0.001, 97.5% CI [−0.43, −0.17]) were associated with lower loneliness.

Next, we examined if adverse childhood experiences, PEE-S, and general trust would predict loneliness. Results demonstrated that the overall regression was significant [*R^2^* = 0.37, *F* (3, 202) = 38.68, *p* < 0.001], and adverse childhood experiences (*β* = 0.27, *p* < 0.001, 97.5% CI [0.14, 0.40]) predicted greater loneliness. In contrast, higher scores in PEE-S (*β* = −0.30, *p* < 0.001, 97.5% CI [−0.43, −0.17]) and general trust (*β* = −0.32, *p* < 0.001, 97.5% CI [−0.45, −0.19]) were related to lower loneliness.

#### 3.2.4 Mediation analyses for happiness (Hypothesis 3)

**Figure 5:**
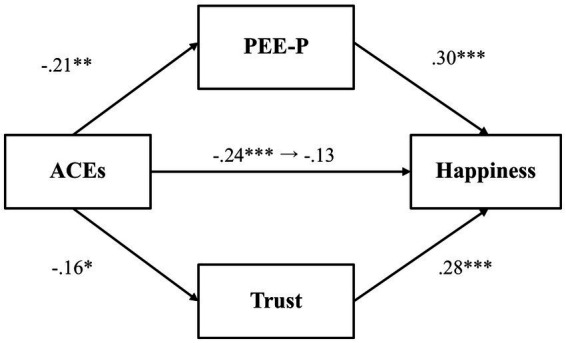
The mediating effect of PEE-P and trust in the link between ACEs and happiness (Study 2). *p < 0.025, **p < 0.01, and ***p < 0.001. ACEs, adverse childhood experiences; PEE-P, positive emotional expression in a personal situation. All values represent standardized coefficients.

A mediation analysis was conducted to explore whether PEE-P and general trust would mediate the relationship between adverse childhood experiences and happiness (Figure 5). Results showed that greater adverse childhood experiences predicted lower PEE-P (*β* = −0.21, *p* = 0.002, 97.5% CI [−0.36, −0.07]), while PEE-P was positively related to happiness (*β* = 0.30, *p* < 0.001, 97.5% CI [0.16, 0.44]). The indirect effect of PEE-P (*β* = −0.06, *p* = 0.01, 97.5% CI [−0.12, −0.01]) was also significant. Adverse childhood experiences predicted general trust as well (*β* = −0.16, *p* = 0.02, 97.5% CI [−0.31, −0.003]), and general trust, in turn, positively predicted happiness (*β* = 0.28, *p* < 0.001, 97.5% CI [0.15, 0.42]), even though the indirect effect of general trust was not significant (*β* = −0.04, *p* = 0.04, 97.5% CI [−0.09, 0.004]). Besides, the total effect (*β* = −0.24, *p* < 0.001, 97.5% CI [−0.38, −0.09]) was significant, whereas the direct effect (*β* = −0.13, *p* = 0.04, 97.5% CI [−0.27, 0.01]) was not, indicating that after accounting for the mediator variables, there was no significant association between adverse childhood experiences and happiness.

**Figure 6:**
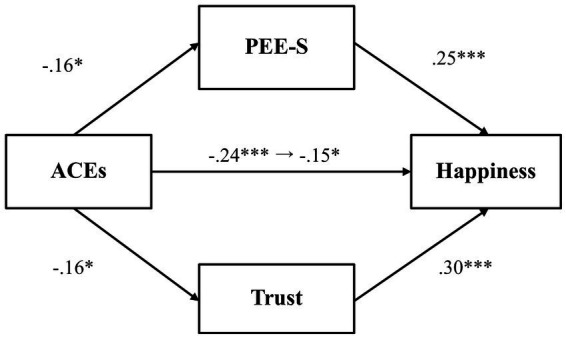
The mediating effect of PEE-S and trust in the link between ACEs and happiness (Study 2). *p < 0.025, **p < 0.01, and ***p < 0.001. ACEs, adverse childhood experiences; PEE-S, positive emotional expression in a social situation. All values represent standardized coefficients.

Next, we investigated whether PEE-S and general trust would mediate the relationship between adverse childhood experiences and happiness (Figure 6). Results indicated that adverse childhood experiences predicted lower PEE-S (*β* = −0.16, *p* = 0.02, 97.5% CI [−0.31, −0.01]), and PEE-S positively predicted happiness (*β* = 0.25, *p* < 0.001, 97.5% CI [0.11, 0.38]), even though the indirect effect of PEE-S (*β* = −0.04, *p* = 0.04, 97.5% CI [−0.08, 0.004]) was not significant. Adverse childhood experiences predicted general trust as well (*β* = −0.16, *p* = 0.02, 97.5% CI [−0.31, −0.003]), and general trust positively predicted happiness (*β* = 0.30, *p* < 0.001, 97.5% CI [0.16, 0.43]), while the indirect effect of general trust was not significant (*β* = −0.05, *p* = 0.04, 97.5% CI [−0.10, 0.004]). The total effect (*β* = −0.24, *p* < 0.001, 97.5% CI [−0.38, −0.09]) and the direct effect (*β* = −0.15, *p* = 0.02, 97.5% CI [−0.29, −0.01]) were both significant, indicating that there was a significant relationship between adverse childhood experiences and happiness even after the influence of mediator variables was held constant.

#### 3.2.5 Mediation analyses for loneliness (hypothesis 4)

**Figure 7:**
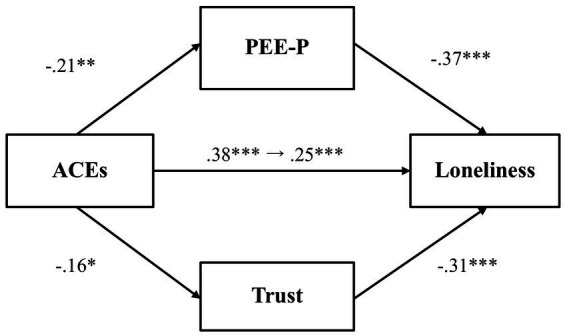
The mediating effect of PEE-P and trust in the link between ACEs and loneliness (Study 2). *p < 0.025, **p < 0.01, and ***p < 0.001. ACEs, adverse childhood experiences; PEE-P, positive emotional expression in a personal situation. All values represent standardized coefficients.

We first explored whether PEE-P and general trust would mediate the relationship between adverse childhood experiences and loneliness (Figure 7). Results demonstrated that adverse childhood experiences predicted lower PEE-P (*β* = −0.21, *p* = 0.001, 97.5% CI [−0.36, −0.07]), and PEE-P was negatively associated with loneliness (*β* = −0.37, *p* < 0.001, 97.5% CI [−0.49, −0.25]). The indirect effect of PEE-P (*β* = 0.08, *p* = 0.01, 97.5% CI [0.02, 0.14]) was also significant. Adverse childhood experiences predicted general trust as well (*β* = −0.16, *p* = 0.02, 97.5% CI [−0.31, −0.003]), and general trust negatively predicted loneliness (*β* = −0.31, *p* < 0.001, 97.5% CI [−0.43, −0.19]), although the indirect effect of general trust was not significant (*β* = −0.05, *p* = 0.04, 97.5% CI [−0.002, 0.10]). The total effect (*β* = 0.38, *p* < 0.001, 97.5% CI [0.25, 0.50]) and the direct effect (*β* = 0.25, *p* < 0.001, 97.5% CI [0.12, 0.37]) were both significant, showing that even after controlling for the mediator variables, there was a significant association between adverse childhood experiences and loneliness.

**Figure 8:**
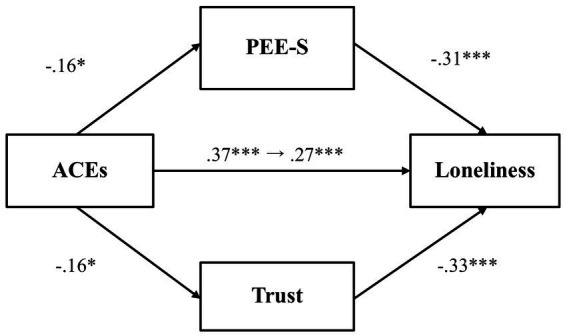
The mediating effect of PEE-S and trust in the link between ACEs and loneliness (Study 2). *p < 0.025, **p < 0.01, and ***p < 0.001. ACEs, adverse childhood experiences; PEE-S, positive emotional expression in a social situation. All values represent standardized coefficients.

Next, the mediating effects of PEE-S and general trust in the link between adverse childhood experiences and loneliness were examined (Figure 8). Results showed that greater adverse childhood experiences predicted lower PEE-S (*β* = −0.16, *p* = 0.02, 97.5% CI [−0.31, −0.01]), and PEE-S, in turn, negatively predicted loneliness (*β* = −0.31, *p* < 0.001, 97.5% CI [−0.43, −0.19]), while the indirect effect of PEE-S (*β* = 0.05, *p* = 0.03, 97.5% CI [−0.002, 0.10]) was not significant. Adverse childhood experiences predicted general trust as well (*β* = −0.16, *p* = 0.02, 97.5% CI [−0.31, −0.003]), and general trust was negatively associated with loneliness (*β* = −0.33, *p* < 0.001, 97.5% CI [−0.45, −0.21]), even though the indirect effect of general trust was not significant (*β* = −0.05, *p* = 0.04, 97.5% CI [−0.002, 0.10]). The total effect (*β* = 0.37, *p* < 0.001, 97.5% CI [0.24, 0.50]) and the direct effect (*β* = 0.27, *p* < 0.001, 97.5% CI [0.15, 0.40]) were both significant, indicating that even after accounting for the mediator variables, adverse childhood experiences significantly predicted higher loneliness.

### 3.3 Discussion

As expected, adverse childhood experiences were generally associated with maladaptive functioning, reflected in lower happiness and greater loneliness. Conversely, PEE-P, PEE-S, and general trust predicted better psychological health, namely, greater happiness and reduced loneliness. Furthermore, PEE-P significantly mediated the relationships between childhood adversity and happiness/loneliness, even though PEE-S and general trust did not.

## 4 Exploratory analyses

Because prior analyses examined American (Study 1) and Japanese participants (Study 2) separately, this exploratory analysis combined the two datasets and tested the hypotheses while accounting for the influence of culture.

### 4.1 Results

#### 4.1.1 The influence of adverse childhood experiences, PEE-P/PEE-S, and trust on happiness, independent of culture

A multiple regression analysis was conducted on happiness, with adverse childhood experiences, PEE-P, general trust, and culture included as independent variables. Results demonstrated that the overall regression was significant [*R^2^* = 0.29, *F* (4, 399) = 40.95, *p* < 0.001]. Adverse childhood experiences were negatively associated with happiness (*β* = −0.13, *p* = 0.003, 97.5% CI [−0.23, −0.03]), even though higher PEE-P (*β* = 0.28, *p* < 0.001, 97.5% CI [0.18, 0.38]) and general trust (*β* = 0.30, *p* < 0.001, 97.5% CI [0.20, 0.40]) predicted greater happiness, independent of culture. Additionally, in a separate analysis where PEE-S was entered instead of PEE-P, the overall regression was also significant [*R^2^* = 0.28, *F* (4, 399) = 38.18, *p* < 0.001]. Adverse childhood experiences were negatively associated with happiness (*β* = −0.16, *p* < 0.001, 97.5% CI [−0.26, −0.06]), while higher PEE-S (*β* = 0.25, *p* < 0.001, 97.5% CI [0.15, 0.36]) and general trust (*β* = 0.30, *p* < 0.001, 97.5% CI [0.20, 0.40]) predicted greater happiness, independent of culture.

#### 4.1.2 The influence of adverse childhood experiences, PEE-P/PEE-S, and trust on loneliness, independent of culture

A multiple regression analysis was conducted on loneliness, with adverse childhood experiences, PEE-P, general trust, and culture included as independent variables. Results indicated that the overall regression was significant [*R^2^* = 0.37, *F* (4, 399) = 57.89, *p* < 0.001]. Adverse childhood experiences predicted greater loneliness (*β* = 0.19, *p* < 0.001, 97.5% CI [0.10, 0.29]), while higher PEE-P (*β* = −0.38, *p* < 0.001, 97.5% CI [−0.48, −0.29]) and general trust (*β* = −0.26, *p* < 0.001, 97.5% CI [−0.35, −0.17]) were associated with lower loneliness, independent of culture. Additionally, in a separate analysis where PEE-S was entered instead of PEE-P, the overall regression was significant [*R^2^* = 0.32, *F* (4, 399) = 47.65, *p* < 0.001]. Adverse childhood experiences (*β* = 0.23, *p* < 0.001, 97.5% CI [0.14, 0.33]) predicted greater loneliness, whereas higher scores in PEE-S (*β* = −0.31, *p* < 0.001, 97.5% CI [−0.42, −0.21]) and general trust (*β* = −0.27, *p* < 0.001, 97.5% CI [−0.37, −0.17]) were related to lower loneliness, independent of culture.

#### 4.1.3 Mediation analyses for happiness

**Figure 9:**
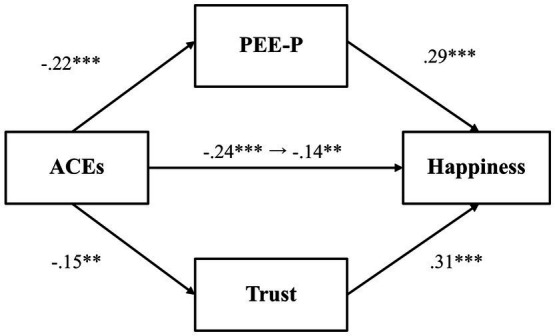
The mediating effect of PEE-P and trust in the link between ACEs and happiness after controlling for culture. **p < 0.01 and ***p < 0.001. ACEs, adverse childhood experiences; PEE-P, positive emotional expression in a personal situation. All values represent standardized coefficients.

A mediation analysis was conducted to explore whether PEE-P and general trust would mediate the relationship between adverse childhood experiences and happiness. Culture was included as a control variable (Figure 9). Results showed that greater adverse childhood experiences predicted lower PEE-P (*β* = −0.22, *p* < 0.001, 97.5% CI [−0.32, −0.11]), and PEE-P was positively related to happiness (*β* = 0.29, *p* < 0.001, 97.5% CI [0.19, 0.38]). Adverse childhood experiences also predicted lower general trust (*β* = −0.15, *p* = 0.002, 97.5% CI [−0.26, −0.04]), and general trust, in turn, positively predicted happiness (*β* = 0.31, *p* < 0.001, 97.5% CI [0.21, 0.40]). The indirect effects of both PEE-P (*β* = −0.06, *p* < 0.001, 97.5% CI [−0.10, −0.03]) and general trust (*β* = −0.05, *p* = 0.01, 97.5% CI [−0.08, −0.01]) were significant. Besides, the total effect (*β* = −0.24, *p* < 0.001, 97.5% CI [−0.35, −0.14]) and the direct effect (*β* = −0.14, *p* = 0.003, 97.5% CI [−0.23, −0.04]) were both significant, indicating that even after accounting for the mediator variables, there remained a significant association between adverse childhood experiences and happiness.

**Figure 10:**
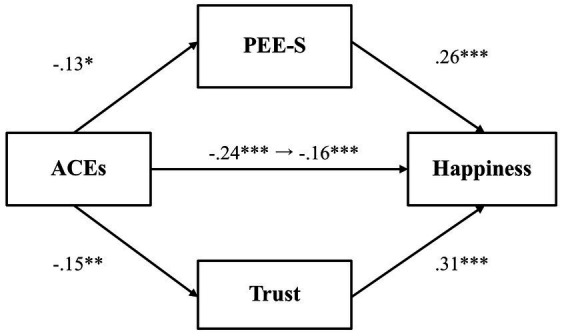
The mediating effect of PEE-S and trust in the link between ACEs and happiness after controlling for culture. *p < 0.025, **p < 0.01, and ***p < 0.001. ACEs, adverse childhood experiences; PEE-S, positive emotional expression in a social situation. All values represent standardized coefficients.

Next, we investigated whether PEE-S and general trust would mediate the relationship between adverse childhood experiences and happiness. Culture was included as a control variable (Figure 10). Results indicated that adverse childhood experiences predicted lower PEE-S (*β* = −0.13, *p* = 0.01, 97.5% CI [−0.24, −0.03]), and PEE-S positively predicted happiness (*β* = 0.26, *p* < 0.001, 97.5% CI [0.16, 0.36]). Adverse childhood experiences predicted general trust as well (*β* = −0.15, *p* = 0.002, 97.5% CI [−0.26, −0.04]), and general trust, in turn, positively predicted happiness (*β* = 0.31, *p* < 0.001, 97.5% CI [0.21, 0.40]). The indirect effects of both PEE-S (*β* = −0.04, *p* = 0.01, 97.5% CI [−0.07, −0.004]) and general trust (*β* = −0.05, *p* = 0.01, 97.5% CI [−0.09, −0.01]) were significant. Moreover, the total effect (*β* = −0.24, *p* < 0.001, 97.5% CI [−0.35, −0.14]) and the direct effect (*β* = −0.16, *p* < 0.001, 97.5% CI [−0.26, −0.06]) were both significant, indicating that there was a significant relationship between adverse childhood experiences and happiness even after the influence of mediator variables was held constant.

#### 4.1.4 Mediation analyses for loneliness

**Figure 11:**
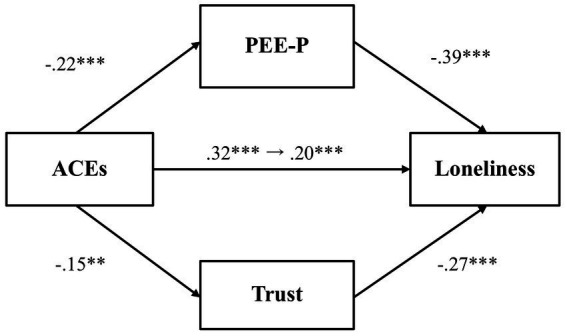
The mediating effect of PEE-P and trust in the link between ACEs and loneliness after controlling for culture. **p < 0.01 and ***p < 0.001. ACEs, adverse childhood experiences; PEE-P, positive emotional expression in a personal situation. All values represent standardized coefficients.

We first explored whether PEE-P and general trust would mediate the relationship between adverse childhood experiences and loneliness, with culture included as a control variable (Figure 11). Results demonstrated that adverse childhood experiences predicted lower PEE-P (*β* = −0.22, *p* < 0.001, 97.5% CI [−0.32, −0.11]), and PEE-P was negatively associated with loneliness (*β* = −0.39, *p* < 0.001, 97.5% CI [−0.48, −0.30]). Adverse childhood experiences predicted general trust as well (*β* = −0.15, *p* = 0.002, 97.5% CI [−0.26, −0.04]), and general trust, in turn, negatively predicted loneliness (*β* = −0.27, *p* < 0.001, 97.5% CI [−0.36, −0.18]). The indirect effects of both PEE-P (*β* = 0.09, *p* < 0.001, 97.5% CI [0.04, 0.13]) and general trust (*β* = 0.04, *p* = 0.01, 97.5% CI [0.01, 0.07]) were significant. Besides, the total effect (*β* = 0.32, *p* < 0.001, 97.5% CI [0.23, 0.42]) and the direct effect (*β* = 0.20, *p* < 0.001, 97.5% CI [0.11, 0.29]) were also significant, showing that even after controlling for the mediator variables, there was a significant association between adverse childhood experiences and loneliness.

**Figure 12:**
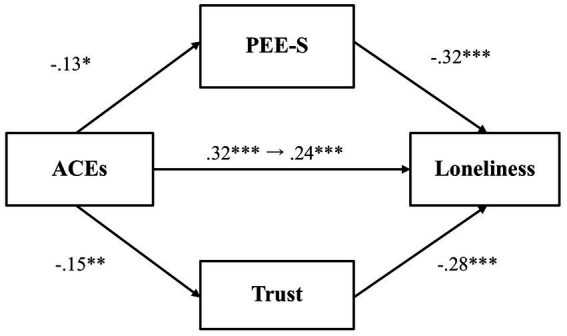
The mediating effect of PEE-S and trust in the link between ACEs and loneliness after controlling for culture. *p < 0.025, **p < 0.01, and ***p < 0.001. ACEs, adverse childhood experiences; PEE-S, positive emotional expression in a social situation. All values represent standardized coefficients.

Next, the mediating effects of PEE-S and general trust in the link between adverse childhood experiences and loneliness were examined, with culture included as a control variable (Figure 12). Results showed that greater adverse childhood experiences predicted lower PEE-S (*β* = −0.13, *p* = 0.01, 97.5% CI [−0.24, −0.03]), and PEE-S, in turn, negatively predicted loneliness (*β* = −0.32, *p* < 0.001, 97.5% CI [−0.42, −0.23]). Adverse childhood experiences also negatively predicted general trust (*β* = −0.15, *p* = 0.002, 97.5% CI [−0.26, −0.04]), and general trust was negatively associated with loneliness (*β* = −0.28, *p* < 0.001, 97.5% CI [−0.37, −0.18]). The indirect effects of both PEE-S (*β* = 0.04, *p* = 0.01, 97.5% CI [0.01, 0.08]) and general trust (*β* = 0.04, *p* = 0.01, 97.5% CI [0.01, 0.08]) were significant. Furthermore, the total effect (*β* = 0.32, *p* < 0.001, 97.5% CI [0.23, 0.42]) and the direct effect (*β* = 0.24, *p* < 0.001, 97.5% CI [0.14, 0.33]) were both significant, indicating that even after accounting for the mediator variables, adverse childhood experiences significantly predicted higher loneliness.

### 4.2 Discussion

Independent of culture, adverse childhood experiences predicted lower happiness and greater loneliness, whereas PEE-P, PEE-S, and general trust were associated with greater happiness and lower levels of loneliness. Besides, PEE-P, PEE-S, and general trust significantly mediated the associations between childhood adversity and happiness/loneliness.

## 5 General discussion

Although a large number of previous studies have documented the detrimental effect of early life adversity on human functioning, the psychological processes underlying this relationship have been relatively underexplored (Cloitre et al., 2019). To fill this gap, the current study aimed to examine the role of positive emotional expression and general trust in the associations between adverse childhood experiences and happiness/loneliness. Study 1, which targeted American participants, showed that adverse childhood experiences were generally associated with lower happiness and greater loneliness, whereas expressing positive emotions in both personal and social situations and general trust predicted greater happiness and lower loneliness. Positive emotional expression in a *personal* situation also mediated the relationships between adverse childhood experiences and happiness/loneliness. Study 2, which extended Study 1 with Japanese participants, demonstrated a similar pattern in which adverse childhood experiences were linked to maladaptive functioning in general (i.e., lower happiness and greater loneliness), while positive emotional expression in both personal and social situations, along with general trust, predicted better psychological states (i.e., higher happiness and lower loneliness). Moreover, as with American participants, positive emotional expression in a personal situation significantly mediated the associations between adverse childhood experiences and happiness/loneliness. Finally, exploratory analyses revealed that the harmful impact of childhood adversity persisted even after controlling for culture, further highlighting the importance of early life experiences for adaptive functioning later in life, regardless of cultural background.

### 5.1 The impact of early life adversity, emotional expression, and trust on health outcomes

Consistent with previous research (e.g., Ohtsubo et al., 2021; Stanton et al., 2000a), childhood adversity was generally associated with lower happiness and greater loneliness among both American and Japanese participants (and after controlling for the influence of culture). Harsh and adverse childhood environments, such as exposure to violence and neglect, disrupt healthy mental and physiological development, leading to lower life satisfaction and wellbeing later in life (Bellis et al., 2013; Mosley-Johnson et al., 2019). Those who have experienced early life adversity are also more likely to struggle with forming and maintaining social connections, which may increase the risk of loneliness and isolation (Merz and Jak, 2013). Therefore, the current findings reinforce and expand upon existing understanding, highlighting that adverse childhood experiences have profound and lasting effects on mental health outcomes across the life course (Kessler et al., 2010).

By contrast, as expected, positive emotional expression in both personal and social situations was associated with higher happiness and lower loneliness irrespective of cultural background. People who express genuine positive emotions, compared to those who do not, tend to experience greater wellbeing because their feelings and behavior align with one another (Mauss et al., 2011). Those who suppress emotions, on the other hand, feel inauthentic themselves due to the discrepancy between their inner feelings and outward expression (English and John, 2013; Mauss et al., 2011), which is costly for both personal health (e.g., lower wellbeing; Kelley et al., 2019) and social functioning (e.g., lower relationship satisfaction, higher loneliness; English and John, 2013; Hirano and Ishii, 2024). Similarly, individuals who express positive emotions when good things happen are seen as authentic by *others* (Mauss et al., 2011). As such, they are more likely to be perceived as trustworthy (Boone and Buck, 2003) and receive greater social support, leading to better psychological functioning (Siedlecki et al., 2014). This is consistent with the findings of Lyubomirsky et al. (2005), indicating that those who express genuine positive emotions are evaluated more favorably (e.g., warm, friendly), thereby expanding their interpersonal relationships. Expressing positive emotions in response to others’ good fortune (i.e., positive emotional expression in a social setting) can also strengthen relational ties and social connections (Gable et al., 2004), ultimately contributing to greater wellbeing (Siedlecki et al., 2014). Moreover, it is worth noting that the happiness of close others is itself predictive of one’s own psychological health (Matsunaga et al., 2017). Overall, the results from the present study support the notion that positive emotional expression, whether in personal or social contexts, plays a crucial role in enhancing adaptive functioning.

As predicted, people with higher levels of general trust reported greater happiness and lower loneliness. Similar to the findings related to emotional expression, this pattern may be in part attributed to the development of positive interpersonal relationships. Because trust can facilitate cooperative behavior, trusting individuals are more likely to have meaningful relationships with others (Tov and Diener, 2009), which is closely linked to human functioning (Siedlecki et al., 2014). However, individuals with lower levels of trust are less likely to seek out or engage in social interactions, thereby increasing the risk of loneliness (Nyqvist et al., 2016). Moreover, loneliness can create a cycle of distrust, where negative assumptions and views about others result in behaviors that push them away, further hindering the formation of supportive relationships (Cacioppo and Hawkley, 2009). Therefore, cultivating trust appears to be one of the critical factors not only in promoting wellbeing but also in breaking the cycle of loneliness.

### 5.2 The mediating effects of positive emotional expression and general trust

Positive emotional expression in a personal situation significantly mediated the relationship between adverse childhood experiences and happiness/loneliness among American and Japanese participants, such that individuals who experienced early life adversity were less likely to express positive emotions when good things happened to them personally, which, in turn, predicted lower happiness and greater loneliness. These relationships can be explained by the impact of early life adversity on self-worth and internal emotional processing. To be more specific, childhood maltreatment often distorts self-concept, leading to feelings of unworthiness and inadequacy (Kim et al., 2022). These negative self-perceptions interfere with the ability to experience or express positive emotions during personal achievements or in personally favorable circumstances, as internal emotional processing is dominated by self-criticism and self-doubt, both of which are linked to depressive symptoms (Kim and Cicchetti, 2006). In other words, negative self-beliefs stemming from childhood adversity hinder the expression of positive emotions in response to personal successes or fortune, thereby increasing the risk of developing psychopathology. Thus, fostering genuine positive emotional expression in personal settings may be a crucial pathway for enhancing happiness and reducing loneliness among those affected by early life adversity.

By contrast, the mediating effects of positive emotional expressivity in a social situation and general trust were not consistently confirmed across the three studies. Specifically, these variables mediated the link between childhood adversity and happiness/loneliness only when American and Japanese datasets were combined (i.e., the mediating effects were non-significant when American and Japanese participants were analyzed individually). Because both constructs are more outward-oriented (i.e., involving interaction or engagement with others), the current findings suggest that early life adversities may exert a stronger influence on more inward-oriented aspects (e.g., positive emotional expression in a *personal* context). However, it is important to note that several studies have documented evidence suggesting the opposite relationship (e.g., Chaturvedi and Arya, 2023). Overall, research exploring these relationships across different cultures remains limited, highlighting the need for further investigation to better understand how childhood maltreatment affects positive emotional expressivity and general trust, as well as their implications for health outcomes.

### 5.3 Limitations of the current study

Our work has several limitations. Firstly, the current study relied solely on retrospective questionnaires, which are often susceptible to recall bias (Reuben et al., 2016). Individuals’ recollections of adverse childhood experiences are also influenced by their current health status, potentially distorting the accuracy of their responses (Colman et al., 2016). In line with this, we did not measure the *present* environments and contextual factors, which might influence the extent to which one expresses positive emotion and trusts others, alongside their utility for overall health. Moreover, the cross-sectional design limits our ability to assess changes over time and determine causal relationships. Therefore, future research should consider the influence of current environments and contextual factors, and adopt longitudinal designs incorporating more objective measures, such as physiological indicators (e.g., cortisol levels or heart rate variability), to enhance the reliability of the findings.

Secondly, the measurement tools used in the current research may not fully capture cultural nuances in “happiness.” In specific, given the emphasis on the *interdependent* self-construal (Markus and Kitayama, 1991), the conceptualization of happiness in the current study (i.e., subjective happiness) may not capture the characteristics of wellbeing among Japanese participants (e.g., greater wellbeing experienced by maintaining relational harmony; Uchida and Ogihara, 2012). Therefore, future investigations should incorporate more culturally sensitive measures (e.g., interdependent happiness scale; Hitokoto and Uchida, 2015) to fully capture the impact of adverse childhood experiences on psychological health across different societies.

Thirdly, the current research focused exclusively on American and Japanese participants. Although existing cross-cultural research has typically classified Western cultures (e.g., the US, Canada) as individualistic and other regions as collectivistic, it is important to recognize that countries from various regions (e.g., Arab countries, Latin America, South Asia) demonstrate varying levels of interdependence (Kitayama et al., 2022) and positive emotional expressivity (Salvador et al., 2024). Therefore, replicating this research with participants from a broader range of cultural backgrounds would be beneficial to ensure the generalizability of the findings.

Finally, the current results should be interpreted with prudence, as measurement invariance for some scales (e.g., PEE-P) was not achieved (see Supplementary Table S2). However, it is worth highlighting that the current study did not necessarily aim to compare cultural differences, but rather sought to explore how the tested hypotheses manifest within different cultural contexts. Besides, although establishing measurement invariance is a commonly sought-after criterion in cross-cultural research, it is important to note that an increasing body of literature challenges the view that it is an absolute requirement for valid interpretation, and that meaningful insights can still be gained even in the absence of full invariance (Robitzsch and Lüdtke, 2023; Welzel et al., 2023; Welzel and Inglehart, 2016). Thus, while the observed non-invariance warrants caution, the current findings still contribute to a nuanced understanding of the tested relationships.

## 6 Conclusion

The present study advances the understanding of psychological mechanisms linking adverse childhood experiences to happiness and loneliness by highlighting the significant role of positive emotional expression in a personal context across cultures. This result underscores the importance of developing therapeutic practices and public health strategies that foster authentic positive emotional expression, particularly in response to personal achievement or fortune. Such approaches can mitigate the lasting effects of childhood maltreatment by empowering affected individuals to reshape their emotional responses, thereby improving long-term mental health and overall wellbeing. Future research should continue to explore additional mediator variables that may underlie the relationships between early life experiences and later-life health outcomes, aiming to develop more targeted and culturally sensitive interventions.

## Data Availability

The raw data supporting the conclusions of this article will be made available by the authors, without undue reservation.

## Footnotes

^1^Nevertheless, we also tested the hypotheses using an aggregated emotional expressivity scale (i.e., combining PEE-P and PEE-S) for reference purposes. For more details, see the Supplementary material.
