# Supplementary material for: Childhood adversity and health: The mediating roles of emotional expression and general trust
Source: Front Psychol. 2024 Nov 20;15:1493421. doi: 10.3389/fpsyg.2024.1493421 (PMC11616180; doi:10.3389/fpsyg.2024.1493421)
Supplement: Supplementary file 1 [file Table_1.pdf]

Supplemental material
Exploratory analysis (combining PEE-P and PEE-S)
Table of Contents

1 Exploratory analyses (Americans) **3**

1.1 Results *3*

1.1.1 The influence of adverse childhood experiences, PEE, and trust on happiness among Americans 3

1.1.2 The influence of adverse childhood experiences, PEE, and trust on loneliness among Americans 3

1.1.3 Mediation analyses for happiness 3

1.1.4 Mediation analyses for loneliness 3

1.2 Discussion *4*

2 Exploratory analyses (Japanese) **4**

2.1 Results *4*

2.1.1 The influence of adverse childhood experiences, PEE, and trust on happiness among Japanese .. 4

2.1.2 The influence of adverse childhood experiences, PEE, and trust on loneliness among Japanese ... 4

2.1.3 Mediation analyses for happiness 4

2.1.4 Mediation analyses for loneliness 5

2.2 Discussion *5*

3 Exploratory analyses with combined data (independent of culture) **5**

3.1 Results *5*

3.1.1 The influence of adverse childhood experiences, PEE, and trust on happiness, independent of culture 5

3.1.2 The influence of adverse childhood experiences, PEE, and trust on loneliness, independent of culture 6

3.1.3 Mediation analyses for happiness 6

3.1.4 Mediation analyses for loneliness 6

3.2 Discussion *6*

Figure S1 .... **8**

Figure S2 .... **9**

Figure S3 .... **10**

Figure S4 .... **11**

Figure S5 .... **12**

Figure S6 .... **13**

4 G*power calculation of sample size with a p-value criterion of .025 .... **14**

Table S1 .... **14**

Table S2 .... **15**

The Subjective Happiness Scale (Lyubomirsky & Lepper, 1999)........ 16

The Emotion Expression Questionnaire (Salvador et al., 2024) 17

The General Trust Scale (Yamagishi et al., 2015)........ 18

The revised UCLA Loneliness Scale (a 20-item scale; Russell et al., 1980) 19

The Risky Family Questionnaire (Taylor et al., 2004) 20

A Japanese version of the Subjective Happiness Scale (Shimai et al., 2004)........ 21

A Japanese version of the Emotion Expression Questionnaire (Salvador et al., 2024)........ 22

A Japanese version of the General Trust Scale (Yamagishi et al., 2015)........ 23

A Japanese version of the revised UCLA Loneliness Scale (a 20-item scale; Moroi, 1992)........ 24

A Japanese version of the Risky Family Questionnaire (Zheng et al., 2020)........ 25

Exploratory analyses (using a combined positive emotion expression scale)

In this exploratory analysis, we combined the scores of positive emotional expression in both personal and social situations to create a composite measure of positive emotional expression (PEE). This approach was taken to gain a more comprehensive understanding of how individuals express positive emotions in general.

1 Exploratory analyses (Americans)

1.1 Results

1.1.1 The influence of adverse childhood experiences, PEE, and trust on happiness among Americans

Multiple regression was conducted to test if adverse childhood experiences, PEE, and general trust would predict happiness. Results demonstrated that the overall regression was significant (R^2 = .29, F (3, 194) = 26.73, p < .001). Adverse childhood experiences were negatively associated with happiness (β = -.15, p = .02, 97.5% CI[-.29, -.01]), even though higher PEE (β = .30, p < .001, 97.5% CI[.15, .44]) and general trust (β = .31, p < .001, 97.5% CI[.16, .45]) predicted greater happiness.

1.1.2 The influence of adverse childhood experiences, PEE, and trust on loneliness among Americans

We first tested if adverse childhood experiences, PEE, and general trust would predict loneliness. Results indicated that the overall regression was significant (R^2 = .32, F (3, 194) = 31.01, p < .001). Adverse childhood experiences predicted greater loneliness (β = .19, p = .002, 97.5% CI[.05, .32]), while higher PEE (β = -.40, p < .001, 97.5% CI[-.54, -.26]) and general trust (β = -.19, p = .002, 97.5% CI[-.33, -.05]) were associated with lower loneliness.

1.1.3 Mediation analyses for happiness

A mediation analysis was conducted to explore whether PEE and general trust would mediate the relationship between adverse childhood experiences and happiness (Figure S1). Results showed that greater adverse childhood experiences predicted lower PEE (β = -.20, p = .01, 97.5% CI[-.35, -.04]), while PEE was positively related to happiness (β = .31, p < .001, 97.5% CI[.17, .44]). The indirect effect of PEE (β = -.06, p = .02, 97.5% CI[-.11, -.01]) was also significant. Adverse childhood experiences, by contrast, did not predict general trust (β = -.15, p = .03, 97.5% CI[-.31, .003]), while general trust positively predicted happiness (β = .31, p < .001, 95% CI[.18, .45]). Besides, the total effect (β = -.26, p < .001, 97.5% CI [-.40, -.11]) and the direct effect (β = -.15, p = .02, 97.5% CI [-.29, -.01]) were both significant, indicating that even after accounting for the mediator variables, there remained a significant association between adverse childhood experiences and happiness.

1.1.4 Mediation analyses for loneliness

We explored whether PEE and general trust would mediate the relationship between adverse childhood experiences and loneliness (Figure S2). Results demonstrated that adverse childhood experiences predicted lower PEE (β = -.20, p = .01, 97.5% CI[-.35, -.04]), and PEE was negatively associated with loneliness (β = -.41, p < .001, 97.5% CI[-.54, -.29]). The indirect effect of PEE (β = .08, p = .01, 97.5% CI[.01, .15]) was also significant. However, adverse childhood experiences did not predict general trust (β = -.15, p = .03, 97.5% CI[-.31, .003]), even though general trust negatively predicted loneliness (β = -.20, p = .002, 97.5% CI[-.33, -.06]). The total effect (β = .30, p < .001, 97.5% CI [.16, .44]) and the direct effect (β = .19, p = .002, 97.5% CI [.06, .33]) were also significant, showing that even after controlling for the mediator variables, there was a significant association between adverse childhood experiences and loneliness.

1.2 Discussion

Consistent with the main findings, adverse childhood experiences predicted lower happiness and greater loneliness, whereas positive emotional expression and general trust predicted greater happiness and lower levels of loneliness. Positive emotional expression also significantly mediated the associations between childhood adversity and happiness/loneliness, although general trust did not.

2 Exploratory analyses (Japanese)

2.1 Results

2.1.1 The influence of adverse childhood experiences, PEE, and trust on happiness among Japanese

Multiple regression was conducted to test if adverse childhood experiences, PEE, and general trust would predict happiness. Results demonstrated that the overall regression was significant (R^2 = .25, F (3, 202) = 22.00, p < .001). Adverse childhood experiences were not significantly associated with happiness (β = -.13, p = .04, 97.5% CI[-.27, .01]), even though higher PEE (β = .29, p < .001, 97.5% CI[.15, .44]) and general trust (β = .27, p < .001, 97.5% CI[.13, .42]) predicted greater happiness.

2.1.2 The influence of adverse childhood experiences, PEE, and trust on loneliness among Japanese

We first tested if adverse childhood experiences, PEE, and general trust would predict loneliness. Results indicated that the overall regression was significant (R^2 = .40, F (3, 202) = 45.00, p < .001). Adverse childhood experiences predicted greater loneliness (β = .25, p < .001, 97.5% CI[.12, .37]), while higher PEE (β = -.37, p < .001, 97.5% CI[-.50, -.24]) and general trust (β = -.30, p < .001, 97.5% CI[-.43, -.17]) were associated with lower loneliness.

2.1.3 Mediation analyses for happiness

A mediation analysis was conducted to explore whether PEE and general trust would mediate the relationship between adverse childhood experiences and happiness (Figure S3). Results showed that greater adverse childhood experiences predicted lower PEE (β = -.20, p = .003, 97.5% CI[-.35, -.05]), while PEE was positively related to happiness (β = .30, p < .001, 97.5% CI[.16, .44]). The indirect effect of PEE (β = -.06, p = .01, 97.5% CI[-.11, -.01]) was also significant. Adverse childhood experiences predicted general trust (β = -.16, p = .02, 97.5% CI[-.31, -.003]), and general trust positively predicted happiness (β = .28, p < .001, 97.5% CI[.14, .41]), while the indirect effect of general trust was not significant (β = -.04, p = .04, 97.5% CI[-.09, .004]). The total effect (β = -.24, p < .001, 97.5% CI [-.38, -.09]) was also significant, while the direct effect (β = -.13, p = .04, 97.5% CI [-.27, .01]) was not, indicating that after accounting for the mediator variables, there was no significant association between adverse childhood experiences and happiness.

2.1.4 Mediation analyses for loneliness

We explored whether PEE and general trust would mediate the relationship between adverse childhood experiences and loneliness (Figure S4). Results demonstrated that adverse childhood experiences predicted lower PEE (β = -.20, p = .003, 97.5% CI[-.35, -.05]), and PEE was negatively associated with loneliness (β = -.38, p < .001, 97.5% CI[-.50, -.26]). The indirect effect of PEE (β = .08, p = .01, 97.5% CI[.02, .14]) was also significant. Adverse childhood experiences predicted general trust (β = -.16, p = .02, 97.5% CI[-.31, -.003]), and general trust negatively predicted loneliness (β = -.31, p < .001, 97.5% CI[-.43, -.19]), although the indirect effect of general trust was not significant (β = .05, p = .04, 97.5% CI[-.002, .10]). The total effect (β = .38, p < .001, 97.5% CI [.25, .50]) and the direct effect (β = .25, p < .001, 97.5% CI [.13, .38]) were also significant, showing that even after controlling for the mediator variables, there was a significant association between adverse childhood experiences and loneliness.

2.2 Discussion

As with American participants, adverse childhood experiences generally predicted lower happiness and greater loneliness, whereas positive emotional expression and general trust were associated with greater happiness and lower levels of loneliness. Moreover, positive emotional expression significantly mediated the associations between childhood adversity and happiness/loneliness, while general trust did not.

3 Exploratory analyses with combined data (independent of culture)

3.1 Results

3.1.1 The influence of adverse childhood experiences, PEE, and trust on happiness, independent of culture

A multiple regression analysis was conducted on happiness, with adverse childhood experiences, PEE, general trust, and culture included as independent variables. Results demonstrated that the overall regression was significant (R^2 = .30, F (4, 399) = 42.37, p < .001). Adverse childhood experiences were negatively associated with happiness (β = -.14, p = .002, 97.5% CI[-.24, -.04]),

even though higher PEE (*β* = .30, $p < .001$, 97.5% CI[.20, .41]) and general trust (*β* = .29, $p < .001$, 97.5% CI[.19, .39]) predicted greater happiness.

3.1.2 The influence of adverse childhood experiences, PEE, and trust on loneliness, independent of culture

A multiple regression analysis was conducted on loneliness, with adverse childhood experiences, PEE, general trust, and culture included as independent variables. Results indicated that the overall regression was significant ($R^2 = .37$, $F\,(4, 399) = 58.21$, $p < .001$). Adverse childhood experiences predicted greater loneliness (*β* = .20, $p < .001$, 97.5% CI[.11, .30]), while higher PEE (*β* = -.39, $p < .001$, 97.5% CI[-.49, -.29]) and general trust (*β* = -.25, $p < .001$, 97.5% CI[-.34, -.15]) were associated with lower loneliness.

3.1.3 Mediation analyses for happiness

A mediation analysis was conducted to explore whether PEE and general trust would mediate the relationship between adverse childhood experiences and happiness, with culture included as a control variable (Figure S5). Results showed that greater adverse childhood experiences predicted lower PEE (*β* = -.19, $p < .001$, 97.5% CI[-.30, -.09]), while PEE was positively related to happiness (*β* = .31, $p < .001$, 97.5% CI[.21, .41]). Adverse childhood experiences also predicted lower general trust (*β* = -.15, $p = .002$, 97.5% CI[-.26, -.04]), and general trust, in turn, positively predicted happiness (*β* = .29, $p < .001$, 97.5% CI[.20, .39]). The indirect effects of PEE (*β* = -.06, $p = .001$, 97.5% CI[-.10, -.02]) and general trust (*β* = -.05, $p = .01$, 97.5% CI[-.08, -.01]) were both significant. Besides, the total effect (*β* = -.24, $p < .001$, 97.5% CI [-.35, -.14]) and the direct effect (*β* = -.14, $p = .002$, 97.5% CI [-.24, -.04]) were both significant, indicating that even after accounting for the mediator variables, there remained a significant association between adverse childhood experiences and happiness.

3.1.4 Mediation analyses for loneliness

We explored whether PEE and general trust would mediate the relationship between adverse childhood experiences and loneliness, with culture included as a control variable (Figure S6). Results demonstrated that adverse childhood experiences predicted lower PEE (*β* = -.19, $p < .001$, 97.5% CI[-.30, -.09]), and PEE was negatively associated with loneliness (*β* = -.40, $p < .001$, 97.5% CI[-.49, -.31]). Adverse childhood experiences predicted general trust as well (*β* = -.15, $p = .002$, 97.5% CI[-.26, -.04]), and general trust, in turn, negatively predicted loneliness (*β* = -.25, $p < .001$, 97.5% CI[-.34, -.16]). The indirect effects of both PEE (*β* = .08, $p < .001$, 97.5% CI[.03, .12]) and general trust (*β* = .04, $p = .01$, 97.5% CI[.01, .07]) were significant. Besides, the total effect (*β* = .32, $p < .001$, 97.5% CI [.23, .42]) and the direct effect (*β* = .21, $p < .001$, 97.5% CI [.12, .30]) were also significant, showing that even after controlling for the mediator variables, there was a significant association between adverse childhood experiences and loneliness.

3.2 Discussion

Independent of culture, adverse childhood experiences predicted lower happiness and greater loneliness, whereas positive emotional expression and general trust predicted greater happiness and lower levels of loneliness. In addition, positive emotional expression and general trust significantly mediated the associations between childhood adversity and happiness/loneliness.

Figure S1

The mediating effect of PEE and trust in the link between ACEs and happiness (Americans)

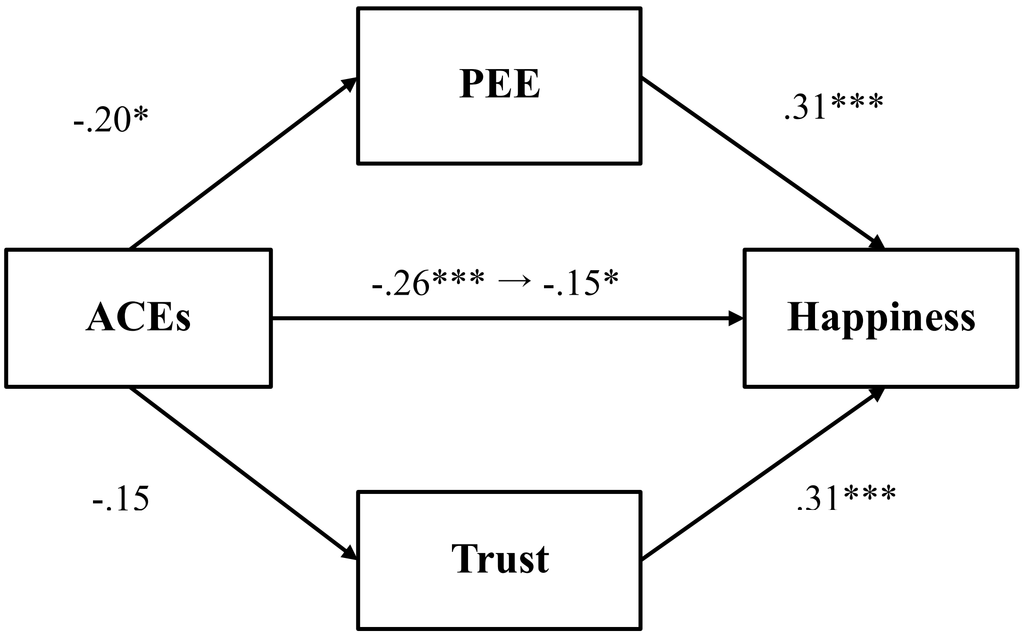

Note. *p < .025. *** p < .001. ACEs = adverse childhood experiences. PEE = positive emotional expression. All values represent standardized coefficients.

Figure S2

The mediating effect of PEE and trust in the link between ACEs and loneliness (Americans)

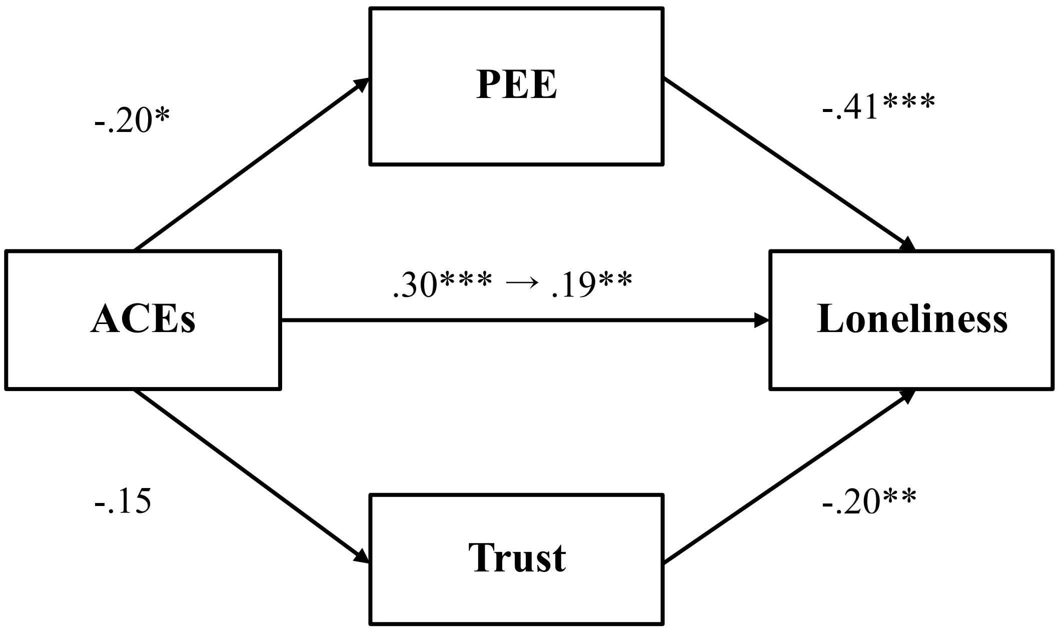

Note. *p < .025. ** p < .01. *** p < .001. ACEs = adverse childhood experiences. PEE = positive emotional expression. All values represent standardized coefficients.

Figure S3

The mediating effect of PEE and trust in the link between ACEs and happiness (Japanese)

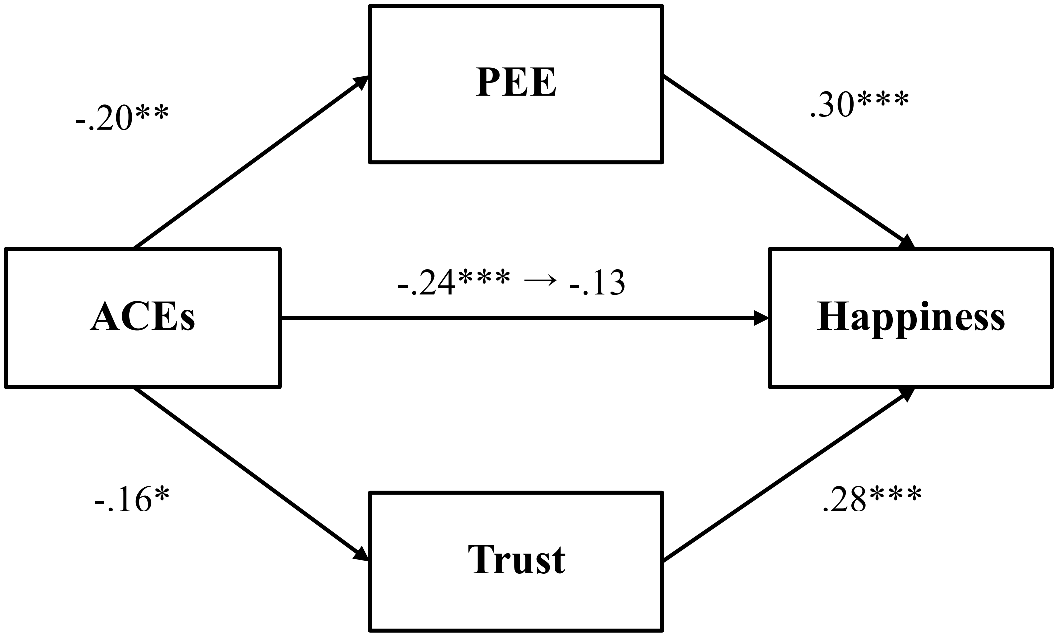

Note. * p < .025. ** p < .01. *** p < .001. ACEs = adverse childhood experiences. PEE = positive emotional expression. All values represent standardized coefficients.

Figure S4

The mediating effect of PEE and trust in the link between ACEs and loneliness (Japanese)

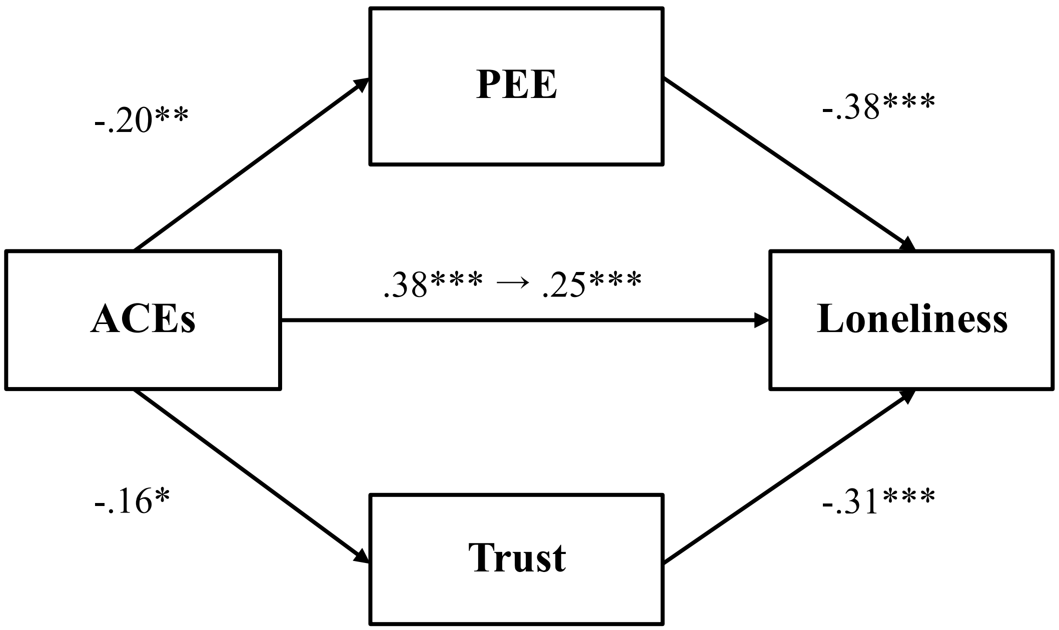

Note. ** $p < .01$. *** $p < .001$. ACEs = adverse childhood experiences. PEE = positive emotional expression. All values represent standardized coefficients.

Figure S5

The mediating effect of PEE and trust in the link between ACEs and happiness after controlling for culture

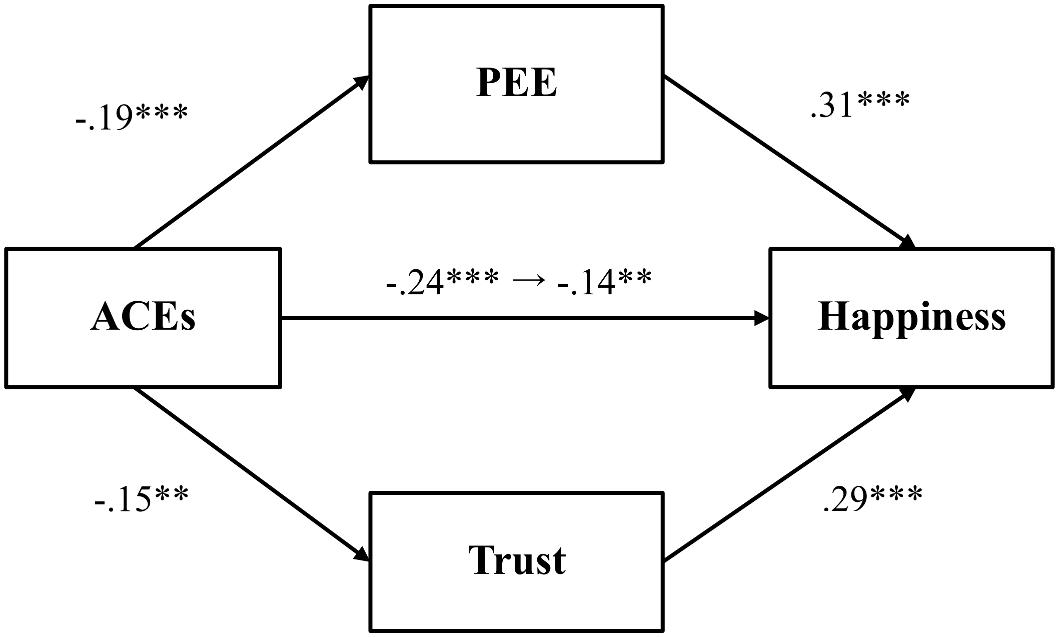

Note. ** $p < .01$. *** $p < .001$. ACEs = adverse childhood experiences. PEE = positive emotional expression. All values represent standardized coefficients.

Figure S6

The mediating effect of PEE and trust in the link between ACEs and loneliness after controlling for culture

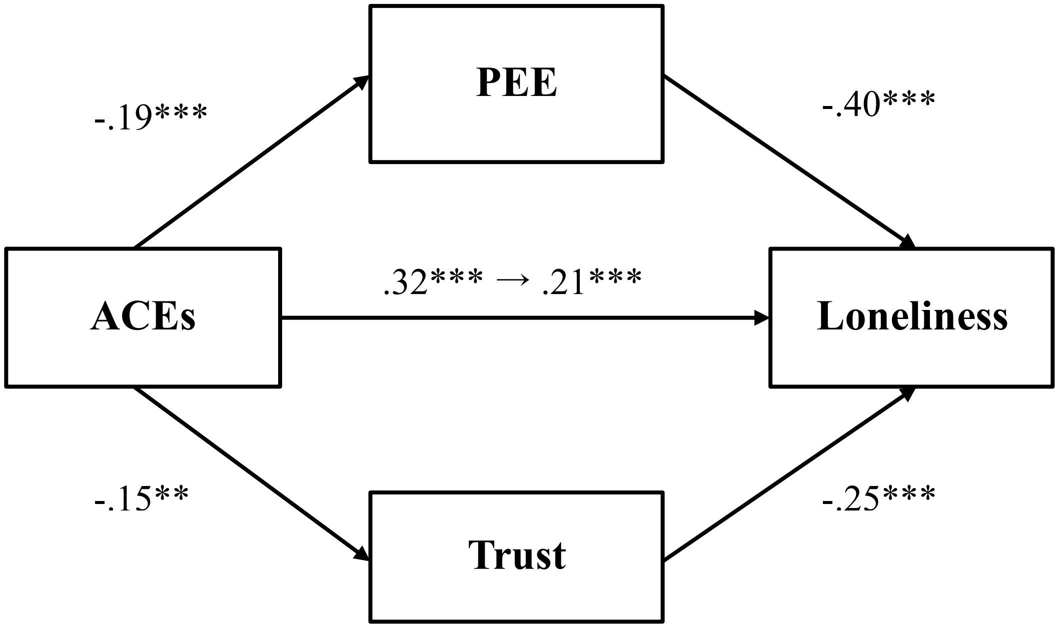

Note. ** $p < .01$. *** $p < .001$. ACEs = adverse childhood experiences. PEE = positive emotional expression. All values represent standardized coefficients.

4 G*power calculation of sample size with a p-value criterion of .025

A power analysis was conducted using G*Power (Faul et al., 2007) to determine the necessary sample size with a p-value criterion of .025. To detect a medium effect size ($f^2 = 0.15$) with 95% power in an F-test (Linear multiple regression: Fixed model, R^2 deviation from zero) with three predictors—childhood family environment, PEE-P or PEE-S, and general trust—and a significance level of .025, a minimum of 136 participants was required. Therefore, the sample sizes of both American (198 participants) and Japanese (206 participants) groups meet this criterion.

Table S1

Skewness and kurtosis values of the variables

	Americans		Japanese	
Variable	Skewness	Kurtosis	Skewness	Kurtosis
ACEs	.32	-1.03	.96	.95
PEE-P	-.69	.21	-.39	.16
PEE-S	-.51	.22	-.52	.42
Trsut	-.32	-.18	-.24	-.57
Happiness	-.13	-.70	.19	-.59
Loneliness	.32	-.62	.01	-.80

Note. ACEs = adverse childhood experiences. PEE-P = positive emotional expression in a personal situation. PEE-S = positive emotional expression in a social situation. The assessment is based on the cut-off values suggested by Hair et al. (2022), indicating that a skewness value between -1 and +1 is excellent and a value between -2 and +2 is acceptable, and a kurtosis value within -2 to +2 is also considered acceptable.

Table S2

Measurement invariance

Measure	Model	*df*	$\chi 2$	CFI	ΔCFI	Invariant
ACEs	Configural	130	745.96***	.750	-	No
PEE-P	Configural	28	199.63***	.861	-	No
PEE-S	Configural	28	81.28***	.951	-	Yes
	Metric	34	90.02***	.948	.003	Yes
Trust	Configural	10	47.12***	.977	-	Yes
	Metric	14	52.19***	.977	.000	Yes
SHS	Configural	4	22.10***	.985	-	Yes
	Metric	7	44.63***	.968	.011	No
	Partial metric	6	33.39***	.977	.008	Yes
Loneliness	Configural	340	1321.94***	.862	-	No

Note. CFI = comparative fit index. ACEs = adverse childhood experiences. PEE-P = positive emotional expression in a personal situation. PEE-S = positive emotional expression in a social situation. *** $p < .001$.

The Subjective Happiness Scale (Lyubomirsky & Lepper, 1999)

Instructions: For each of the following statements and/or questions, please select the point on the scale that you feel is most appropriate in describing you.

Q1. In general, I consider myself:

1. Not a very happy person ~ 7. A very happy person

Q2. Compared with most of my peers, I consider myself:

1. Less happy ~ 7. More happy.

Q3. Some people are generally very happy. They enjoy life regardless of what is going on, getting the most out of everything. To what extent does this characterization describe you?

1. Not at all ~ 7. A great deal

Q4. Some people are generally not very happy. Although they are not depressed, they never seem as happy as they might be. To what extent does this characterization describe you?

1. Not at all ~ 7. A great deal

The Emotion Expression Questionnaire (Salvador et al., 2024)

Instructions: In this part of the study, we will ask you what emotions would you express in different social situations. By expressing emotions we mean using gestures or facial expressions to show how you feel. Read each social situation carefully and then think how strongly you would express different emotions when you are discussing the situation with your friends and family members.

Situation 1. When you have succeeded in an exam or assignment.

How strongly would you express each emotion below when you discuss your experience with your friends or family members?

1. Feelings of closeness to others
2. Elated
3. Self-esteem
4. Happy
5. Calm
6. Proud
7. Friendly feelings

Situation 2. When you learned about something good that happened to your friends or family.

How strongly would you express each of the 12 emotions below when you discuss your experience with your friends or family members?

1. Feelings of closeness to others
2. Elated
3. Self-esteem
4. Happy
5. Calm
6. Proud
7. Friendly feelings

The General Trust Scale (Yamagishi et al., 2015)

Instructions: Each item is a statement that a person may either agree with or disagree with. For each item, indicate how much you agree or disagree with what the item says.

1. Most people are basically honest.
2. Most people are basically good-natured and kind.
3. Most people trust others.
4. Generally, I trust others.
5. Most people are trustworthy.

The revised UCLA Loneliness Scale (a 20-item scale; Russell et al., 1980)

Instructions: Indicate how often each of the statements below is descriptive of you.

1. I feel in tune with the people around me.
2. I lack companionship.
3. There is no one I can turn to.
4. I do not feel alone.
5. I feel part of a group of friends.
6. I have a lot in common with the people around me.
7. I am no longer close to anyone.
8. My interests and ideas are not shared by those around me.
9. I am an outgoing person.
10. There are people I feel close to.
11. I feel left out.
12. My social relationships are superficial.
13. No one really knows me well.
14. I feel isolated from others.
15. I can find companionship when I want it.
16. There are people who really understand me.
17. I am unhappy being so withdrawn.
18. People are around me but not with me.
19. There are people I can talk to.
20. There are people I can turn to.

The Risky Family Questionnaire (Taylor et al., 2004)

Instructions: These are questions about when you were growing up (between ages 5-15). Please think over your family life and answer these questions. If you feel uncomfortable answering any of these items, you may skip them and proceed to the next question or section.

1. How often did a parent or other adult in the household make you feel that you were loved, supported, and cared for?
2. How often did a parent or other adult in the household swear at you, insult you, put you down, or act in a way that made you feel threatened?
3. How often did a parent or other adult in the household express physical affection for you, such as hugging, or other physical gestures of warmth and affection?
4. How often did a parent or other adult in the household push, grab, shove, or slap you?
5. How often would you say that a parent or other adult in the household behaved violently toward a family member or visitor in your home?
6. How often would you say there was quarreling, arguing, or shouting between your parents?
7. How often would you say there was quarreling, arguing, or shouting between a parent and you?
8. How often would you say there was quarreling, arguing, or shouting between a parent and one of your siblings?
9. How often would you say there was quarreling, arguing, or shouting between your sibling(s) and you?
10. Would you say the household you grew up in was chaotic and disorganized?
11. In your childhood, did you live with anyone who was a problem drinker or alcoholic, or who used street drugs?
12. Would you say that the household you grew up in was well-organized and well-managed?
13. Would you say you were neglected while you were growing up, left on your own to fend for yourself?

A Japanese version of the Subjective Happiness Scale (Shimai et al., 2004)

質問：以下の４項目についてあなたが自分に当てはまると思う数字を選択し回答してください。

Q1. 一般的に見て、あなたご自身の幸福度はどのレベルにあてはまりますか？ 7段階で評価してください。

1. 全く幸せではない～7. とても幸せだ

Q2. 大半の友人や同僚と比べて、あなたご自身の幸福度はどのレベルにあてはまりますか？ 7段階で評価してください。

1. 全く幸せではない～7. とても幸せだ

Q3. 概してとても幸せだという人たちがいます。そのような人たちは、どのような状況にあっても人生を楽しみ、あらゆるものから最高のものをつかみ取る人たちです。あなたはこのような特徴にどれくらいあてはまりますか？7段階で評価してください。

1. 全く当てはまらない～7. かなり当てはまる

Q4. 概してあまり幸せでないという人たちがいます。そのような人たちは抑うつではないのですが、幸せであるはずなのに決して幸せそうには見えない人たちです。あなたはこのような特徴にどれくらいあてはまりますか？7段階で評価してください。

1. 全く当てはまらない～7. かなり当てはまる

A Japanese version of the Emotion Expression Questionnaire (Salvador et al., 2024)

ここでは、さまざまな社会的状況においてあなたがどのような感情を示すのかについてお尋ねします。感情表出とは、あなたがどのように感じているかをジェスチャーや顔の表情を使って示すことを意味します。注意深くそれぞれの社会的状況を読み、あなたが友達や家族とその状況について話すときにどの程度強くさまざまな感情を示すのかを答えていただきます。

状況 1. あなたが試験や与えられた課題で成功したとき

あなたがその状況での経験を友達や家族に話すとき、以下の感情をどの程度強く表出しますか？

1. ふれあい
2. うきうき
3. 自尊感情
4. 幸せ
5. 落ち着き
6. 誇り
7. 親しみ

状況 2 あなたが友達または家族に良いことが起きたことを知ったとき

あなたがその状況での経験を友達や家族に話すとき、以下の 12 の感情のそれぞれをどの程度強く表出しますか？

1. ふれあい
2. うきうき
3. 自尊感情
4. 幸せ
5. 落ち着き
6. 誇り
7. 親しみ

A Japanese version of the General Trust Scale (Yamagishi et al., 2015)

質問：次の文章について、あなた自身の考えに最も近い数字を選択してください。

1. ほとんどの人は基本的に正直である
2. ほとんどの人は基本的に善良 (ぜんりょう) で親切である
3. ほとんどの人は他人を信頼している
4. 私は人を信頼するほうである
5. ほとんどの人は信用できる

A Japanese version of the revised UCLA Loneliness Scale (a 20-item scale; Moroi, 1992)

質問：あなたは、以下の文章のようなことをどれくらい感じますか。

1. 私は、自分の周囲の人たちと調子よくいっている。
2. 私は、人とのつきあいがない。
3. 私には、頼りにできる人がだれもいない。
4. 私は、ひとりぼっちではない。
5. 私は、親しい仲間たちのなかで欠くことのできない存在である。
6. 私は、自分の周囲の人たちと共通点が多い。
7. 私は、今、だれとも親しくしていない。
8. 私の興味や考えは、私の周囲の人たちとはちがう。
9. 私は、外出好きの人間である。
10. 私には、親密感のもてる人たちがいる。
11. 私は、無視されている。
12. 私の社会的なつながりはうわべだけのものである。
13. 私をよく知っている人はだれもいない。
14. 私は、他の人たちから孤立している。
15. 私は、望むときにはいつでも、人とつきあうことができる。
16. 私には、私を本当に理解してくれる人たちがいる。
17. 私は、たいへん引っ込み思案なのでみじめである。
18. 私には、知人はいるが、私と同じ考えの人はいない。
19. 私には、話しかけることのできる人たちがいる。
20. 私には、頼りにできる人たちがいる。

A Japanese version of the Risky Family Questionnaire (Zheng et al., 2020)

質問：以下の項目では、あなたが子供の頃（5 歳～15 歳）についてうかがいます。あなたの家族生活を思い出して、以下の質問に回答してください。また、答えたくないと思った質問には回答せず、先に進んでいただいて構いません。

1. 親（または同じ家庭で生活していた他の成人）から愛されている、支えてもらっている、大事に思われていると感じることがどれくらいの頻度でありましたか？
2. 親（または同じ家庭で生活していた他の成人）があなたを罵ったり、侮辱したり、あなたに馬鹿にしたり、あなたを怖がらせるようなふるまいをすることがどれくらいの頻度でありましたか？
3. 親（または同じ家庭で生活していた他の成人）があなたに身体的な愛情表現（たとえばあなたをハグしたり、その他のあたたかな感情や愛情をジェスチャーで示すこと）がどれくらいの頻度でありましたか？
4. 親（または同じ家庭で生活していた他の成人）があなたを突飛ばしたり、強くつかんだり、乱暴に押したり、平手でうったりすることがどれくらいの頻度でありましたか？
5. 親（または同じ家庭で生活していた他の成人）が家族のだれか、または家に尋ねてきた人に対して暴力的に振る舞うことがどれくらいの頻度でありましたか？
6. あなたの両親が口げんかをしたり、口論をしたり、大声で言い争いをすることがどれくらいの頻度でありましたか？
7. あなたとあなたの親（両親のうちどちらか）が口げんかをしたり、口論をしたり、大声で言い争ったりすることがどれくらいの頻度でありましたか？
8. あなたの親（両親のうちどちらか）とあなたのきょうだいの誰かが口げんかをしたり、口論をしたり、大声で言い争ったりすることがどれくらいの頻度でありましたか？
9. あなたのきょうだいとあなたが口げんかをしたり、口論をしたり、大声で言い争ったりすることがどれくらいの頻度でありましたか？
10. あなたが育った家庭は混乱して無秩序な状態だったと思いますか？
11. あなたが子供の頃、お酒を飲んで問題を起こす人もしくはアルコール依存症の人、またはドラッグを使用している人と一緒に住んでいたことがありましたか？
12. あなたが育った家庭は整理整頓されていて管理がいきとどいていましたか？
13. あなたが子どもの頃、あなたはネグレクトされ、自分自身で身の回りのことをしなければならないような状態であったことがありますか？
